# Directional Second Sphere Effect on CO_2_ Activation and Reduction by Iron Porphyrin in Aprotic and Protic Media

**DOI:** 10.1002/anie.1490828

**Published:** 2026-07-23

**Authors:** Ashutosh Vishwakarma, Sami Essid, Sarah Bouery, Régis Guillot, Bernard Boitrel, Philipp Gotico, Marie Sircoglou, Zakaria Halime

**Affiliations:** ^1^ CNRS Institut De Chimie Moléculaire Et Des Matériaux d'Orsay Université Paris‐Saclay Orsay France; ^2^ CNRS ISCR (Institut Des Sciences Chimiques De Rennes)‐UMR 6226 Univ Rennes Rennes France; ^3^ Institute For Integrative Biology of the Cell CEA CNRS Université Paris‐Saclay Gif‐sur‐Yvette France

**Keywords:** carbon dioxide, directionality, electrocatalysis, hydrogen‐bond, second coordination sphere

## Abstract

The topological directionality of functional groups in the secondary coordination sphere (SCS) plays a decisive role in enzymatic small‐molecule activation but remains underexplored in synthetic systems. Here, we investigate how symmetry remodeling of a urea‐based SCS modulates electrocatalytic CO_2_‐to‐CO reduction using two atropisomeric iron porphyrins, ***αβαβ*‐UrFe** and ***α_2_β_2_*‐UrFe**, which differ solely in the spatial arrangement of identical hydrogen‐bond donor groups. Switching from a face‐to‐face to an adjacent urea‐arms configuration enables unprecedented CO_2_‐to‐CO conversion under strictly aprotic conditions, in the absence of Brønsted or Lewis acids. Combined electrochemical, spectroscopic, kinetic, and DFT studies reveal that the adjacent positioning of the urea arms weakens the stabilization of the initial [Fe─CO_2_] intermediate but creates a more open and dynamically reorganizable SCS that permits insertion of a second CO_2_ molecule as an oxygen‐atom acceptor, to yield CO and CO_3_^2^ˉ. Under protic conditions, this improved active‐site accessibility, combined with a dissymmetrization of the activated CO_2_ molecule, accelerates proton transfer, with CO_2_‐to‐CO reduction reaching turnover frequency of up to 2.72 × 10^7^ s^−1^, among the highest reported for homogeneous catalysis. These results establish directional interactions within the SCS as powerful design strategy for boosting molecular catalysis.

## Graphical abstract

Directional control within the secondary coordination sphere redefines CO_2_ reduction by iron porphyrins. Simply rearranging identical urea hydrogen‐bond donors modulates reactivity, revealing that optimal catalysis does not only arise from maximal intermediate stabilization, but also from directional, adaptive non‐covalent interactions that balance binding and reactivity.

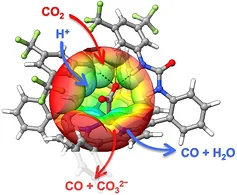

## 1 Introduction

The activation and redox transformation of small, relatively inert atmospheric molecules such as CO_2_, O_2_, and N_2_ under mild conditions represents one of the most fundamental and challenging goals in both biological and energy‐related synthetic chemistry [1]. Metalloenzymes have evolved highly efficient strategies to activate these molecules under ambient conditions by leveraging both the primary coordination environment and the surrounding protein matrix [2, 3, 4, 5, 6, 7, 8, 9]. In particular, the second coordination sphere (SCS) plays a critical role in modulating reactivity through non‐covalent interactions, hydrogen bonding networks, and electrostatic fields. In contrast to synthetic catalysts, which often adopt idealized high‐symmetry geometries when incorporating multiple SCS functional groups due to synthetic constraints [10, 11, 12, 13], metalloenzymes feature lower‐symmetry SCS architectures that play an important role in modulating electronic structure, redox properties, and substrate binding affinities. Enzymes such as cytochrome P450s [14, 15], carbon monoxide dehydrogenases [2, 5, 16, 17], and nitrogenases [8, 9] exemplify the functional advantages of low‐symmetry active sites. These configurations favor directional interactions with highly symmetric substrates such as CO_2_, O_2_, and N_2_, allowing their controlled activation by dissymmetrization to favor the formation of polar intermediates through dynamic interactions [18].

Several recent studies have systematically examined how the topological positioning of SCS functional groups influences the electrocatalytic CO_2_ reduction reaction (CO2RR) by synthesizing distinct atropisomers within the porphyrin catalyst family (Scheme 1). In 2020, our group reported that the *αα* and *αβ* atropisomers of an iron porphyrin bearing two urea groups (Scheme 1a) catalyze CO2RR at comparable overpotentials, yet with markedly higher catalytic rates for the *αβ* atropisomer [19]. Experimental and computational evidences suggest that although CO_2_ binding is more favored in the *αα* configuration, the *αβ* allows a more efficient proton access to the distal oxygen of the mono‐urea activated CO_2_, facilitating C─O bond cleavage. In contrast, Zhang and Cao later reported a catalytic superiority for the *αα* atropisomer of iron porphyrins bearing trimethylbenzylamine substituents compared to the *αβ* analog (Scheme 1b). DFT calculations indicate that this arrangement allows one group to interact with the CO_2_ adduct while the other engages the proton source via electrostatic interactions. Likewise, Jiang and co‐workers observed enhanced catalytic activity for the *αα* atropisomer of zinc porphyrins featuring triazole units (Scheme 1c), which are proposed to facilitate proton transfer from the hydrophilic network of the bulk solvent to the CO_2_‐bound intermediate [20]. In 2021, Mayer and co‐workers reported that the orientation of cationic trimethylanilinium groups in iron porphyrins is optimal in the *αααα* atropisomer (Scheme 1d) which achieves the highest CO2RR rate but at the cost of the highest overpotential [21]. The correlation between turnover frequency (TOF) and overpotential points to an electronic scaling effect driven by the electron‐withdrawing nature of the cationic substituents [22], while the enhanced catalytic rates likely arise from favorable through‐space electrostatic interaction, consistent with previous observations for imidazolium‐based cationic moieties [23]. In heterogeneous catalysis, the group of Lu, Peng, and Zhang exploited Cu porphyrin atropisomers bearing salicylimine groups (Scheme 1e), where the *αααα* atropisomer favors CO formation whereas the *αβαβ* atropisomer favors CH_4_ production when heterogenized on a carbon‐based electrode [24]. Collectively, these studies highlight the potential of atropisomerism as a design principle for tuning the electrocatalytic activity and selectivity of porphyrin complexes by modulating substrate binding, activation, and proton co‐substrate delivery [25]. Furthermore, studies involving hydrogen‐bond donors typically compare atropisomers featuring varying numbers of functionalities within each SCS site (*e.g*., *αααα vs αβαβ, αα vs αβ*). A systematic approach would thus be essential to disentangle the effects of positioning and directionality from those related to the number of SCS functionalities involved in the reaction.

**SCHEME 1:**
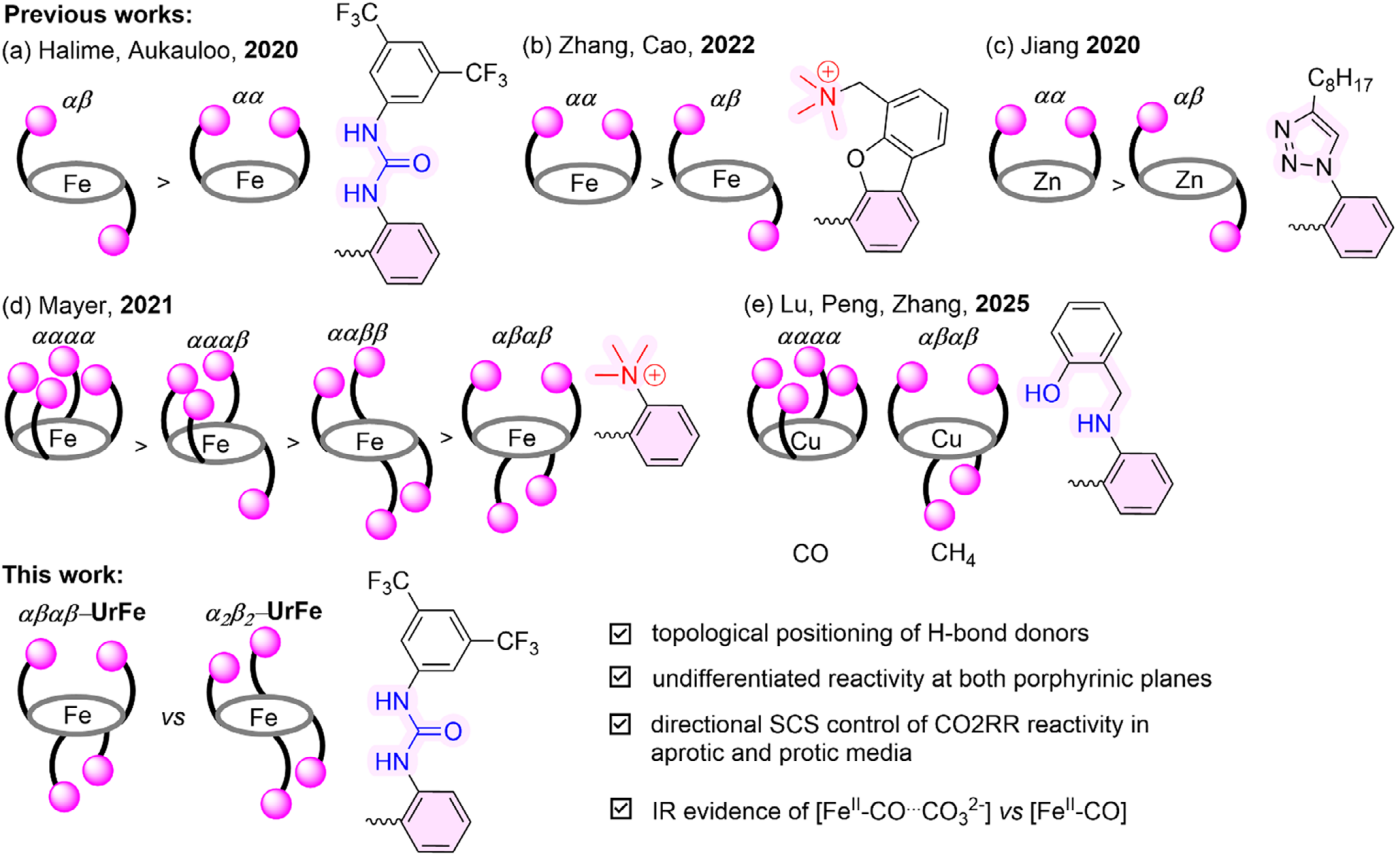
Various atropisomeric porphyrin catalysts reported for the electrocatalytic CO_2_ reduction that explore topological positioning (pink) of H‐bond donors (blue) and cationic groups (red). Pink spheres represent the substituents introduced at the meso‐phenyl groups described next to the topologies. Symbol ‘>’ represents the relative catalytic rate enhancement.

In this work, we leverage a unique opportunity arising from the geometric differences between the *α_2_β_2_* and *αβαβ* atropisomers of a urea‐functionalized iron porphyrin catalyst to investigate the impact of SCS symmetry on the catalytic performances in the electrochemical CO_2_‐to‐CO reduction. We previously reported a D_2d_ symmetric ***αβαβ*‐UrFe** catalyst [26], with two urea groups acting as multipoint H‐bond donors positioned in a face‐to‐face configuration on each side of the porphyrin macrocycle (Scheme 2). In contrast, the ***α_2_β_2_*‐UrFe** catalyst was designed with a lower C_2h_ symmetry, where the two urea groups on each side of the porphyrin are located at adjacent meso positions. These systems present a rare and controlled case in which both catalysts bear identical numbers and types of functional groups in the SCS, differing only in their spatial arrangement, with each exhibiting identical SCS on both faces of the porphyrin ring. This structural configuration provides an ideal platform to probe how SCS directionality modulates the balance between CO_2_ capture, stabilization, and reactivity, ultimately influencing the catalytic activity for CO_2_ reduction. Remarkably, the ***α_2_β_2_*‐UrFe** catalyst demonstrates an unprecedented ability to catalyze this reaction under aprotic media, without any external proton source or Lewis acid, producing CO and CO_3_^2^ˉ. Under protic conditions, switching from the ***αβαβ*** to the ***α_2_β_2_*** configuration boosts turnover frequency (TOF) by three orders of magnitude, achieving, to the best or our knowledge, one of the highest reported rates for homogeneous CO_2_‐to‐CO electrocatalysis (*k*_cat_ = 2.72 × 10^7^ s^−1^) by an iron‐porphyrin‐based catalyst. Below, we present comprehensive electrochemical, kinetic, spectroscopic, and computational studies that reveal how this subtle tuning of SCS directionality modulates CO_2_ activation while dynamically reorganizing the SCS to accommodate co‐substrates such as water and a second CO_2_ molecule as oxide acceptor, thereby dictating the observed reactivity differences.

**SCHEME 2:**
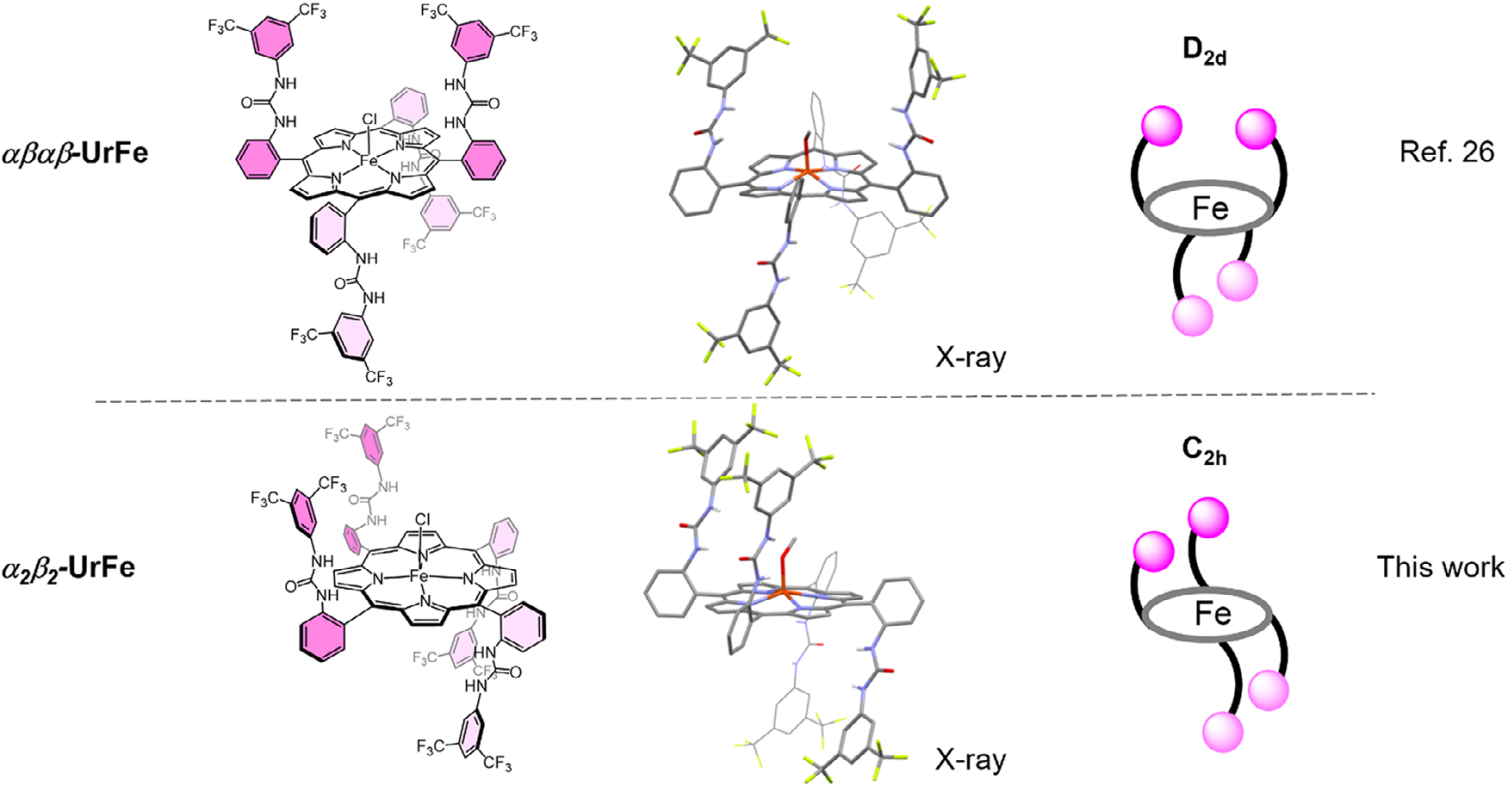
Molecular drawing of ***αβαβ*‐UrFe** (top left) and ***α_2_β_2_*‐UrFe** (bottom left) and the wireframe and capped sticks representation of their corresponding x‐ray structures (***αβαβ*‐UrFe‐OMe**: top center, CCDC 1884364; ***α_2_β_2_‐*UrFe‐OH**: bottom center, CCDC 2536618) with a methoxide anion as an axial ligand. Only urea protons are explicitly shown, and solvent molecules are omitted for clarity. Schematic symmetry representation of ***αβαβ*‐UrFe** (top right) and ***α_2_β_2_‐*UrFe** (bottom right).

## 2 Results and Discussion

The ***α_2_β_2_‐*UrFe** catalyst was synthesized by adapting our previously reported synthetic strategy for ***αβαβ*‐UrFe** [26]. All synthetic procedures (Figure S1) and characterizations (Figures S24–S30) are gathered in the Supporting Information (SI). The retention of the initial porphyrin geometry throughout the multistep synthesis of the ligand was first confirmed by nuclear magnetic resonance (NMR) spectroscopy (Figures S26–S28), which indicated the exclusive presence of a single atropisomer. Further confirmation of the three‐dimensional structure of ***α_2_β_2_*‐UrFe** was obtained via single‐crystal X‐ray diffraction, using crystals grown by slow evaporation of a saturated solution in a mixture of acetone, methanol, and water (Scheme 2 and Figure S23; Table S8 provides detailed crystallographic parameters). All catalysts studied here were used in their Fe(III)─Cl state with a chloride axial ligand. However, during the crystallization process for single‐crystal X‐ray diffraction analysis, the Cl^ˉ^ ligand was displaced by methoxide (from methanol) in the case ***αβαβ*‐UrFe** and hydroxide (from water) in the case of ***α_2_β_2_*‐UrFe**. Comparison of the solid‐state structures of ***α_2_β_2_*‐UrFe** with that of ***αβαβ*‐UrFe** highlights the influence of porphyrin symmetry on the spatial arrangement of the urea groups in the SCS. In ***αβαβ*‐UrFe**, the two urea moieties are positioned in an almost linear arrangement with an angle of 162° between the carbon atoms of the urea groups and the axial oxygen atom bound to the iron center. In contrast, ***α_2_β_2_*‐UrFe** exhibits a significantly more acute angle of 106° between the equivalent atoms. This geometric divergence shows that the symmetry of the porphyrin core significantly modulates the spatial orientation of the SCS functional groups, potentially leading to different interaction modes with the linear CO_2_ substrate.

To investigate the effect of this orientation difference on the electrocatalytic properties of the urea modified iron–porphyrin for CO_2_‐to‐CO reduction, the cyclic voltammogram (CV) of ***α_2_β_2_*‐UrFe** (1 mM) was first recorded in argon (Ar)‐purged, anhydrous dimethylformamide (DMF) containing 0.1 M of tetra‐*N*‐butylammonium hexafluorophosphate ([Bu_4_N]PF_6_) as the supporting electrolyte. As expected for an iron porphyrin, three reversible redox waves corresponding to the formal Fe(III/II), Fe(II/I), and Fe(I/0) redox events were observed (Figure 1a), with the latter two more precisely assigned as Fe^II^Por/Fe^II^Por^‒^ and Fe^II^Por/Fe^II^Por^2‒^, respectively. While the metal‐centered Fe(III/II) reduction exhibits minimal variation between the two atropisomers, the ligand‐based reductions show more pronounced differences, with ***α_2_β_2_*‐UrFe** displaying a 60 mV cathodic shift in the formal Fe(II/I) couple and 90 mV cathodic shift in the formal Fe(I/0) couple relative to ***αβαβ*‐UrFe** (Table S1). This shift is surprising, as both catalysts experience the same inductive electron‐withdrawing influence from the four urea arms. The calculated diffusion coefficients (Table S3) indicate a smaller hydrodynamic size of ***αβαβ*‐UrFe** (D = 1.40 × 10^−6^ cm^2^ s^−1^) compared to ***α_2_β_2_*‐UrFe** (D = 0.69 × 10^−6^ cm^2^ s^−1^), which could result from a more compact arrangement of the urea arms in solution (Figure S33) thereby facilitating its reduction on the electrode surface.

**FIGURE 1:**
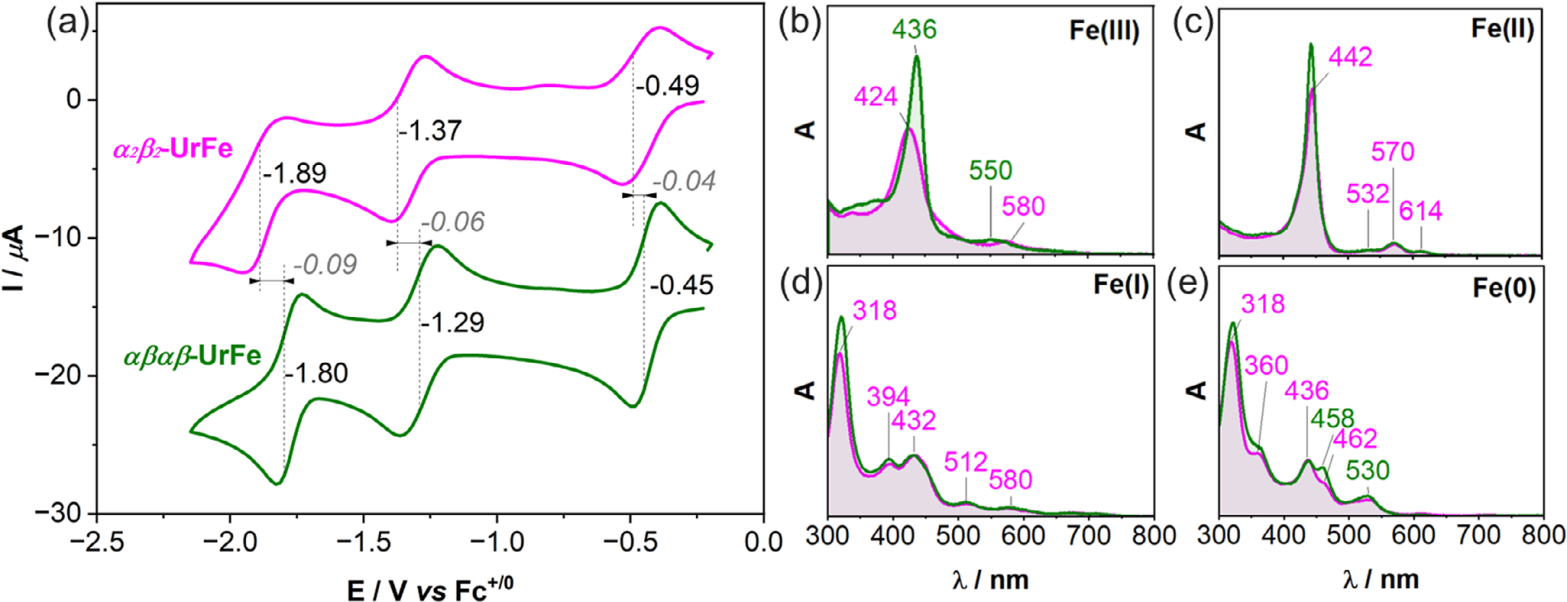
(a) Cyclic voltammograms of 1 mM ***α_2_β_2_*‐UrFe** (pink) and ***αβαβ*‐UrFe** (green) under Ar‐saturated dry DMF containing 0.1 M [Bu_4_N]PF_6_ at 25°C, showing *E*_1/2_ redox potentials (black) and the potential differences between the two atropisomers (gray). (b–d) Spectral characteristics of the reduced states [Fe(III), Fe(II), Fe(I) and Fe(0)] of the iron porphyrin catalysts in N_2_‐saturated DMF containing 0.1 M [Bu_4_N]PF_6_. Spectral evolution for each of the catalyst are detailed in Figures S16 and S17.

UV–vis spectroscopy was employed to probe potential electronic differences between the two atropisomers that could account for the observed variations in redox potentials. In their ground‐state Fe(III)–Cl form (Figure 1b), the iron porphyrin catalysts display different spectral features. Notably, the Soret band shifts from 436 to 424 nm and the Q band from 550 to 580 nm when comparing ***αβαβ*‐UrFe** to ***α_2_β_2_*‐UrFe**. At this oxidation state, these differences likely arise from distinct interaction modes with the axial chloride ligand, depending on the relative positioning of the urea groups, which may induce subtle distortions of the porphyrin macrocycle. Upon reduction to the Fe(II) state, accompanied by chloride dissociation, and further to the Fe(I) and Fe(0) states, no significant spectral differences are observed between the atropisomers (Figure 1c–e). These findings further suggest that the electrochemical discrepancies are unlikely to originate from intrinsic electronic differences, but rather from electrode–molecule interfacial interactions that are not captured under homogeneous spectroscopic conditions.

When the reaction atmosphere was switched from Ar to CO_2_, a more striking difference between the two catalysts emerged. As previously reported for ***αβαβ*‐UrFe**, this transition induces an anodic shift in the Fe(I/0) redox wave (Figure 2a), attributed to an early CO_2_ binding to the Fe(I) species facilitated by hydrogen‐bonding interactions within the SCS [27]. A similar but more pronounced anodic shift (120 mV) is also observed here for ***α_2_β_2_*‐UrFe**; however, in this case, the redox wave is now immediately followed by a marked increase in the current intensity (Figure 2b). Because such a behavior is unprecedented for iron porphyrins in absence of a proton source or a Lewis acid, bulk electrolysis (BE) experiment coupled to gas chromatography (GC) was performed to determine whether this current increase corresponds to the electrocatalytic CO_2_ reduction (Figures S14, Table S4). These experiments unambiguously confirm the generation of CO, albeit with a modest Faradaic efficiency (FE) of 29%, upon applying a potential of −1.86 V vs Fc^+^^/0^.

**FIGURE 2:**
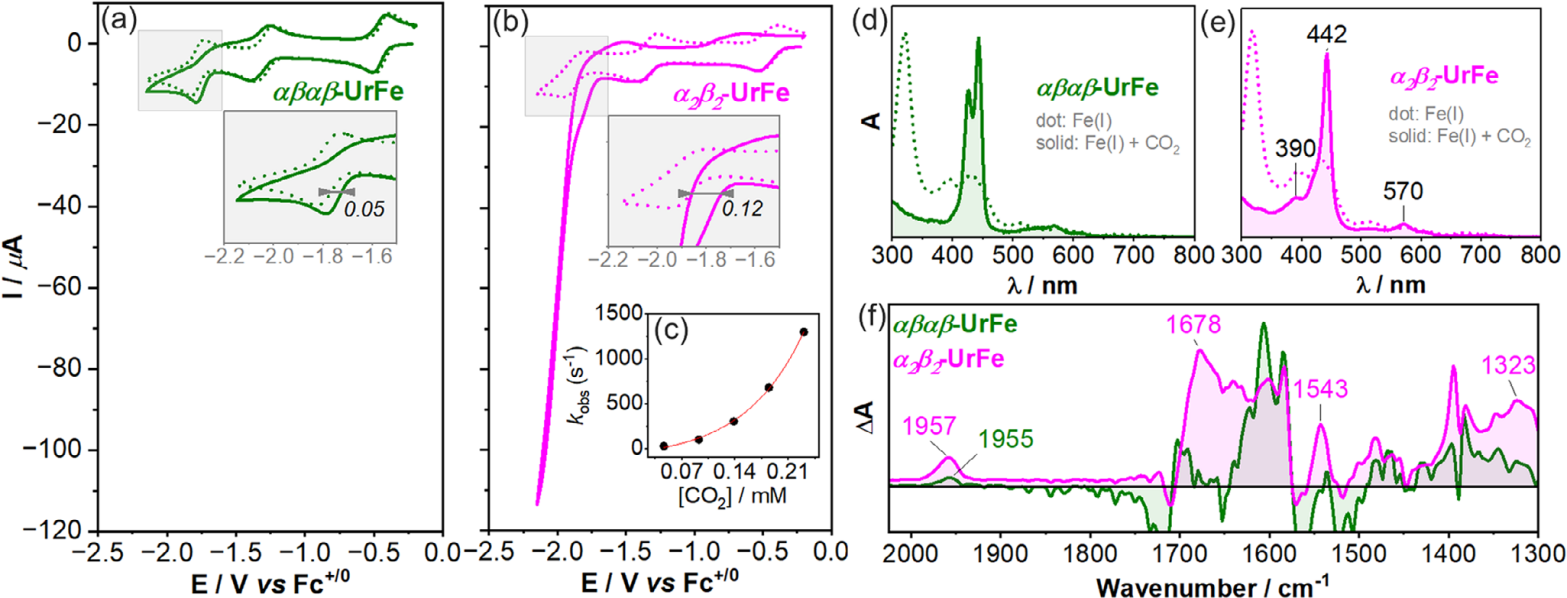
(a) Cyclic voltammograms of 1 mM ***αβαβ*‐UrFe** (green) and (b) ***α_2_β_2_*‐UrFe** (pink) under Ar‐saturated (dotted line) and CO_2_‐saturated (solid line) dry DMF containing 0.1 M [Bu_4_N]PF_6_ at 25°C. Insets show a zoom on the anodic shift of the Fe(I/0) redox wave upon CO_2_ interaction. (c) Dependence of the catalytic rate, estimated by foot‐of‐the‐wave (FOW) analysis, on the concentration of CO_2_ for the ***α_2_β_2_*‐UrFe** catalyst (Figure S9 for more details). UV–vis spectral features of (d) ***αβαβ*‐UrFe** (green) and (e) ***α_2_β_2_*‐UrFe** (pink) for the Fe(I) state in the absence (dotted lines) and presence of CO_2_, obtained by thin‐layer spectroelectrochemistry in DMF (spectral evolution in Figures S18 and S19). (f) IR spectral features at an applied potential of –2.05 V *vs* Fc^+/0^ comparing ***αβαβ*‐UrFe** (green) and ***α_2_β_2_*‐UrFe** (pink) using thin‐layer spectroelectrochemistry of CO_2_‐saturated deuterated acetonitrile (spectral evolution in Figures S21).

Beyond the classical proton‐assisted CO_2_‐to‐CO electroreduction pathway (CO_2_ + 2H^+^ + 2e^– ^→CO + H_2_O), Savéant and co‐workers have reported an alternative mechanism operating under aprotic conditions. In this case, two molecules of CO_2_ are involved, one being reduced into CO while the second serves as an oxygen atom acceptor (2 CO_2_ + 2e^– ^→ CO + CO_3_^2–^) in the presence of an activating exogenous Lewis acid (Mg^2+^, Ca^2+^, Ba^2+^, Li^+^ or Na^+^) [28, 29]. Foot‐of‐the‐wave (FOW) analyses of the CVs [30, 31, 32, 33] under varying CO_2_ concentrations (Figure 2c) reveals a rate dependence consistent with second‐order kinetics, supporting the involvement of two CO_2_ molecules in the catalytic cycle. A similar mechanism therefore likely operates here, but driven by the topological configuration of the urea arms in the ***α_2_β_2_*‐UrFe**. UV‐Visible spectroelectrochemistry (UV–vis SEC) indicates CO_2_ already binds at the Fe(I) state for both atropisomers forming [**Fe^III^‐CO_2_]^–^**, with distinct spectral features. For ***αβαβ*‐UrFe** (Figure 2d), new bands appear at 424, 442, and 568 nm, while ***α_2_β_2_*‐UrFe** exhibits features at 390, 442, and 570 nm (Figure 2e), reflecting differences in their electronic environments. Baseline drift during measurements at more negative potential unfortunately limited the characterization of the [**Fe^II^‐CO_2_]^2–^** species by UV–Vis SEC. Infrared spectroelectrochemistry (IR SEC) at the catalytic potential of –2.05 V vs Fc^+/0^ in dry deuterated acetonitrile (Figure 2f) additionally revealed new IR bands for ***α_2_β_2_*‐UrFe** at 1678, 1957, 1543 and 1323 cm^−1^, that were computationally assigned to an **[Fe^II^‐CO…CO_3_Ur]^2–^** and **[Fe^I^‐CO_3_]** ^3−^) intermediates (vide infra and Table S14).

To account for such a reactivity, the mechanism of the aprotic reduction of CO_2_ was investigated computationally using density functional theory (DFT). Calculations employed simplified models denoted **^m^ *αβαβ‐*UrFe** and **^m^ *α_2_β_2_*‐UrFe** in which the two urea arms on one face of the porphyrin were replaced with protons. The geometry of each intermediate was optimized at the CPCM(DMF)‐D3‐OLYP/def2svp level of theory and its energy was refined at SMD(DMF)‐D3(BJ)‐OLYP/def2tzvpp (see Supporting Information for detailed procedures). The coordination of the first CO_2_ molecule was confirmed to be accessible from the formal oxidation state Fe(I) and is calculated to be more favored for **^m^ *αβαβ‐*UrFe(I)** compared to **^m^ *α_2_β_2_*‐UrFe(I)** (–6.6 vs + 2.5 kcal.mol^−1^). However, a further one‐electron reduction of these species is required prior to reaction with a second CO_2_ molecule (Table S11). Structural analysis of the resulting Fe─CO_2_ intermediates reveals the formation of hydrogen bonds between the urea arms and the bound CO_2_ in both atropisomers (Figure 3a, b). As previously described for **^m^ *αβαβ‐*UrFe**, the CO_2_ is symmetrically clamped by two sets of hydrogen bonds between each O atom and one urea group (Figure 3a, c). In contrast, in **^m^ *α_2_β_2_*‐UrFe**, the two urea groups interact with the same oxygen atom via three H‐bonds in a dissymmetrical fashion (Figure 3b, d). The binding of CO_2_ is less stabilizing in this intermediate compared to the previous example (–11.8 vs –20.8 kcal.mol^−1^), but this could be mainly due to the removal of one H‐bond. Interestingly, these mono‐directional interactions activate the CO_2_ fragment to a comparable extent to that in **^m^ *αβαβ‐*UrFe**, according to calculated CO_2_ bending (Figure S33) and neat charge (Table S12). This suggests that the redesign of the SCS in **^m^ *α_2_β_2_*‐UrFe** does not alter significantly the intrinsic basicity of the Fe─CO_2_ intermediate. However, a marked difference is found in the accessibility of the distal O atom which is much higher in the case of **^m^ *α_2_β_2_‐*UrFe**. The buried volume analysis indeed reveals significantly lower V_bur_ values for **^m^ *α_2_β_2_*‐UrFe** than **^m^ *αβαβ‐*UrFe**, both in the accessible quadrant (32.4% vs 60.0%) and octant (6.4% vs 33.6%) decompositions (Figure 3e, f).

**FIGURE 3:**
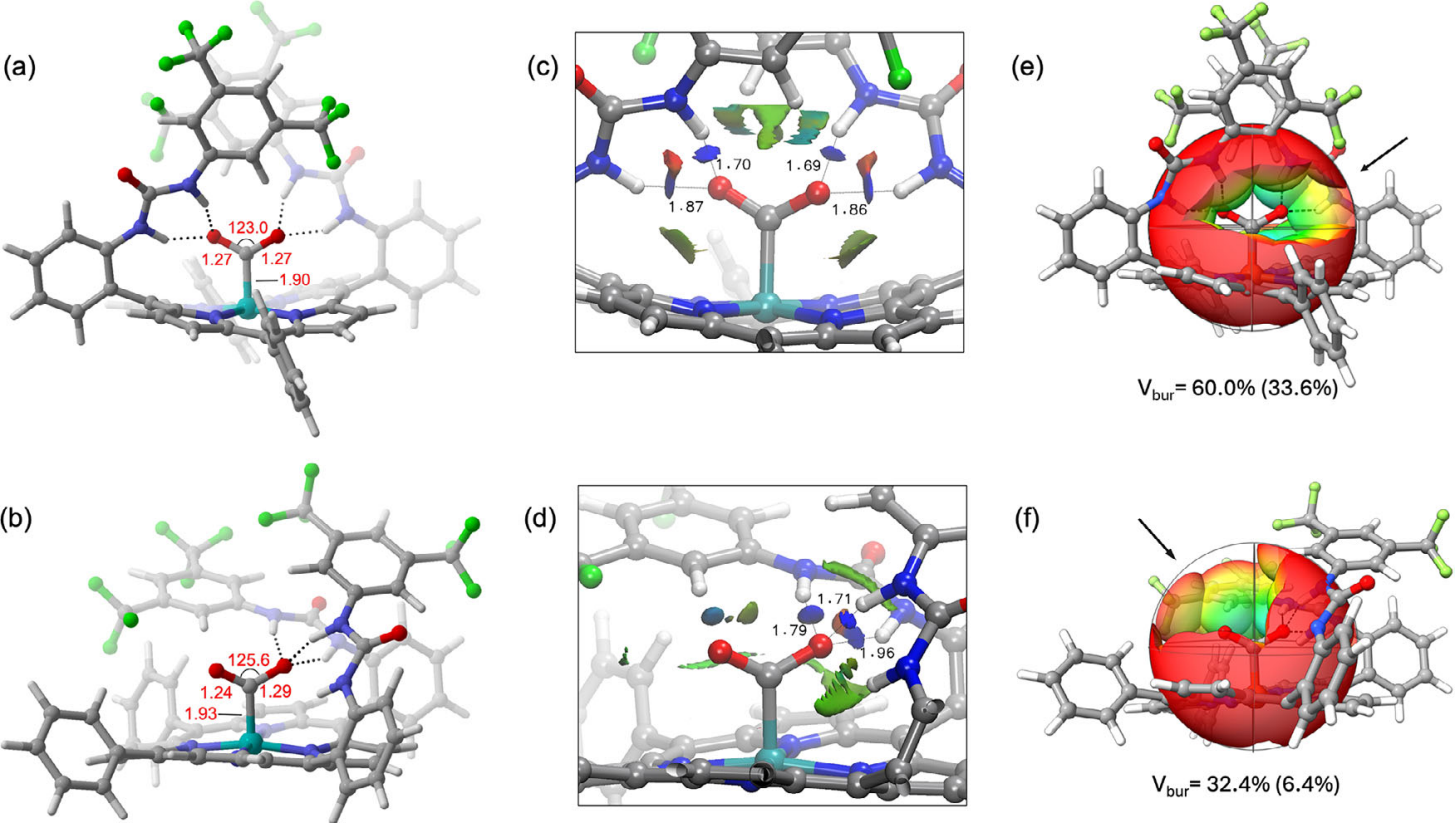
Comparison of the symmetrical *vs* dissymmetrical activation of CO_2_ in the tri‐reduced state of the two modeled atropisomers **[^m^ *αβαβ*‐UrFe‐CO_2_]^2–^** (*top*) and **[^m^ *α_2_β_2_*‐UrFe‐CO_2_]^2–^** (*bottom*). (a and b) Optimized structures with relevant bond distances (Å) and angles (degree). (c and d) Non‐covalent interaction (NCI) plots between the CO_2_ fragment and the iron porphyrin. The color of the surfaces indicates the nature of the interactions with red = strongly repulsive, green = very weak, blue = strongly attractive. The hydrogen bonds are characterized by the presence of deep blue lentils. (e and f) Buried volume around the CO_2_ fragment calculated within a 4 Å sphere centered at the carbon, VDW scale of 1.17, with Fe─C taken as *z*‐axis and the plane containing CO_2_ as *xz*‐plane. The hindrance brought by the Fe atom is excluded for clarity. The color scale indicates the distance from the C center (blue = closest, red = farthest). The percentage of buried volume (V_bur_) are given for the upper quadrant (upper front octant) pointed by an arrow at the most accessible oxygen atom, which corresponds to the direction of the C─O bond formation.

As a result, the **[^m^ *α_2_β_2_*‐UrFe─CO_2_]^2–^** intermediate enables the insertion of a second CO_2_ molecule to form the bis‐CO_2_ adduct (Figure S36) via a moderately endergonic but kinetically accessible step (∆G = +6.2 kcal.mol^−1^, ∆G^‡^ = +7.5 kcal.mol^−1^). Notably, the O─C bond formation between the two CO_2_ fragments is accompanied by a rearrangement of the hydrogen‐bonding network, with the singly H‐bonded urea shifting from the Fe‐coordinated CO_2_ to the incoming CO_2_ molecule (Figure 4). In contrast, the insertion of a second CO_2_ molecule onto the symmetrically clamped mono CO_2_ adduct within **^m^ *αβαβ*‐UrFe** is thermodynamically unfavorable (∆G = +17.4 kcal.mol^−1^), and would yield a substantially distorted Fe─(CO_2_)_2_ (Figure S34, Table S13). In addition, its formation would require the decoordination of a tightly bound urea arm and the insertion of the second CO_2_ molecule in a more sterically buried Fe─CO_2_ site (Figure 3e) and no transition state could be found to account for this transformation. Surprisingly, the second CO_2_ insertion to the non‐functionalized **[TPPFe─CO_2_]^2–^** where the lack of SCS interactions is offset by a greater Fe─CO_2_ accessibility require a comparable cost to those of **^m^ *α_2_β_2_*‐UrFe** (barrierless process, ∆G = +7.3 kcal.mol^−1^, Figure S34). However, subsequent C─O bond cleavage to carbonate is prohibitively endergonic without a Lewis acid (+30.1 kcal.mol^−1^). In contrast, in **^m^ *α_2_β_2_*‐UrFe**, hydrogen‐bonding transiently stabilizes the departing carbonate, lowering the activation barrier to 16.0 kcal.mol^−1^ (Figures 4, S35 and S36). The resulting **[Fe^II^** ─**CO…CO_3_‐Ur]^2–^** intermediate exhibits calculated vibrational signatures at 1948 and 1680 cm^−1^ (Figure 4) corresponding to the C─O stretches at the Fe‐bound carbon monoxide and the urea‐bound carbonate, respectively, in excellent agreement with the experimental IR SEC observations (Figure 2f).

**FIGURE 4:**
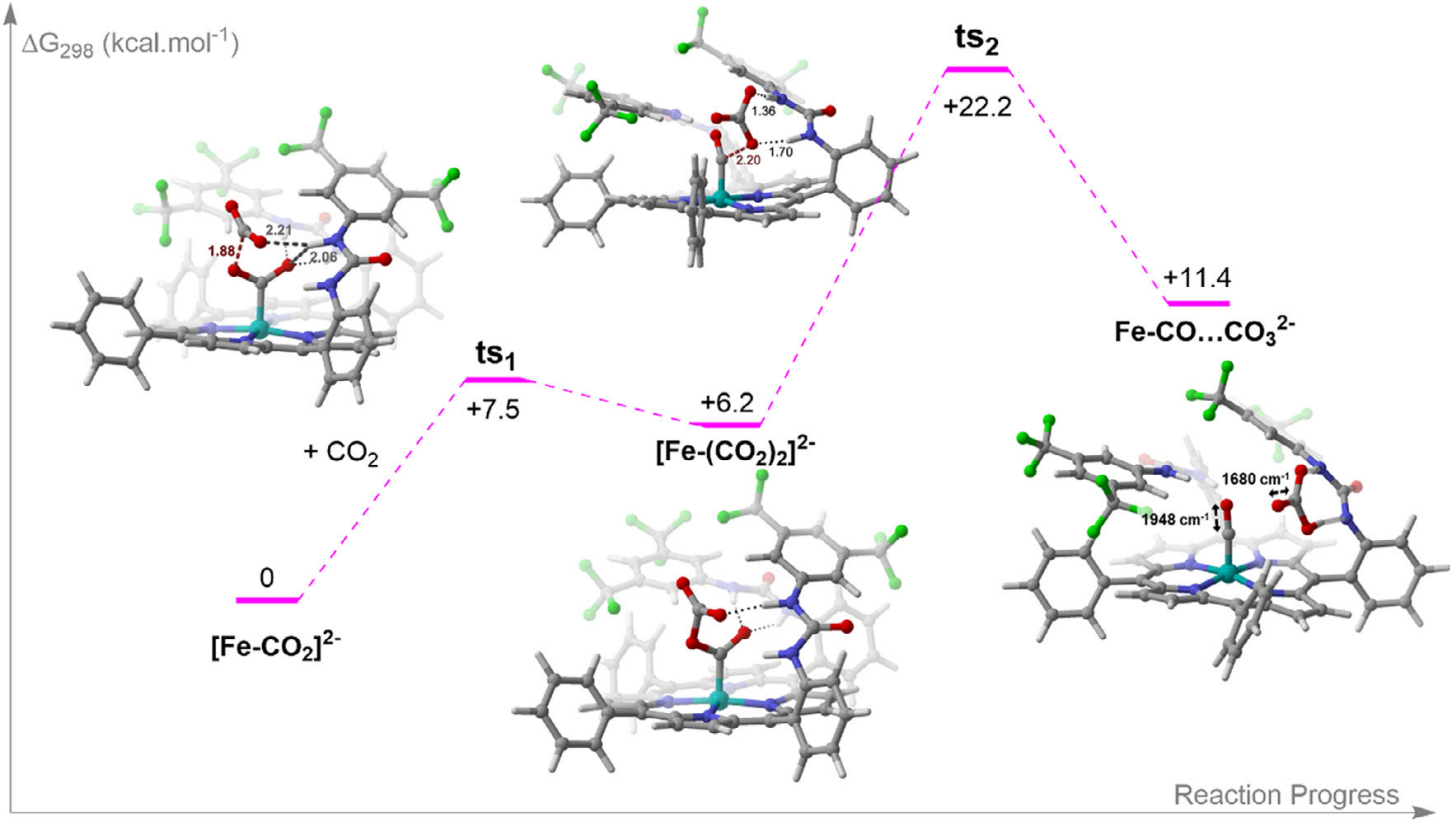
Gibbs free energy profile calculated at the CPCM(DMF)‐D3‐OLYP/def2svp//SMD(DMF)‐D3(BJ)‐OLYP/def2tzvpp level of theory, showing the formation of CO and carbonate at **^m^ *α_2_β_2_*‐UrFe**, following the formation of the [Fe─CO_2_]^2−^ adduct. Bond breaking/forming at transition states are shown as dashed lines. Distances are indicated in Å. Relevant vibrational modes involving CO and carbonate stretches are given for intermediate **[Fe^II^** ─**CO…CO_3_‐Ur]^2–^**.

The unprecedented ability of ***α_2_β_2_*‐UrFe** to catalyze CO_2_ electroreduction without exogenous Brønsted or Lewis acids distinguish it from the ***αβαβ*‐UrFe** atropisomer. The elements brought by our mechanistic study indicates that the SCS of ***α_2_β_2_*‐UrFe** is optimally positioned to participate in each step of the aprotic CO2RR mechanism, from CO_2_ binding and activation, to the functionalization of the distal O atom, and its abstraction to form CO. This optimal design also extends to protic CO2RR as shown by a direct comparison of the catalytic performances of ***αβαβ*‐UrFe** (Figure 5a) and ***α_2_β_2_*‐UrFe** (Figure 5b) in the presence of 5.55 M of water as a proton source. Under these conditions, both catalysts exhibit important catalytic waves; however, ***α_2_β_2_*‐UrFe** shows significantly higher current intensity, and a more pronounced anodic shift of the onset potential (210 mV shift compared to 150 mV) indicative of higher activity at lower overpotential. CO was identified as the exclusive product, with a FE of 84%, as determined by BE coupled to GC (Figure S14, Table S4), and no liquid products were detected. The corresponding log(TOF_max_) versus overpotential (*η*) plots obtained from FOW analysis for these two catalysts is shown in Figure 5c, together with benchmark data for the non‐functionalized iron tetraphenylporphyrin (**TPPFe**), its perfluorinated analogues (**F_8_Fe, F_20_Fe**), the four trimethylanilinium‐modified iron porphyrin atropisomers (***αααα*** *‐*, ***α_3_β*** *‐*, ***α_2_β_2_‐*** *, and* ***αβαβ‐*FeTMA**) reported by Mayer, and our previously reported ***αα‐*** and ***αβ***‐**UrFe** (see Table S7 and Figure S22 for full comparison with the state‐of‐the‐art) [21, 26, 34]. This comparison clearly illustrates that the incorporation of urea groups as hydrogen‐bond‐donating SCS motifs in ***α_2_β_2_*‐UrFe** and ***αβαβ*‐UrFe** enables these catalysts to break away from the linear scaling trend imposed by purely through‐bond electron‐withdrawing effect of the fluorine substituents, which typically enforce a trade‐off between lower overpotentials and reduced TOFs. Moreover, the atropisomeric effect appears to exert a much stronger influence with urea‐based H‐bond‐donating groups than with previously studied cationic substituents. This trend underscores that beyond CO_2_ binding and activation, site accessibility and hydrogen‐bond donor directionality are critical design elements.

**FIGURE 5:**
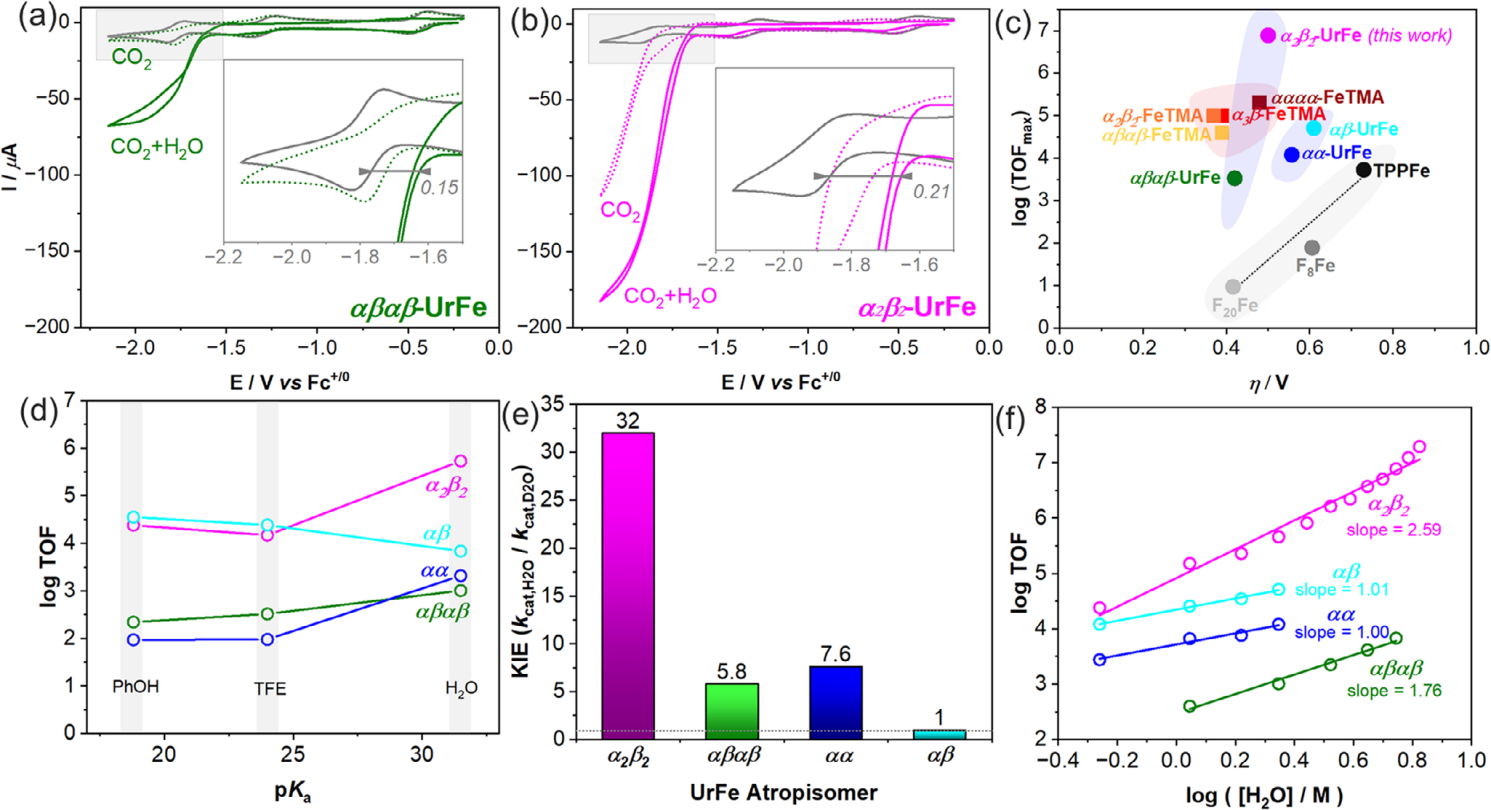
Cyclic voltammograms of 1 mM (a) ***αβαβ*‐UrFe** (green) and (b) ***α_2_β_2_*‐UrFe** (pink) under argon‐ (gray), CO_2_‐saturated (dotted lines) dry DMF containing 0.1 M [Bu_4_N]PF_6_ at 25°C and in presence of 5.55 M of H_2_O (solid lines). (c) Comparison of log(TOF_max_) versus overpotential *(η*) for ***α_2_β_2_*‐UrFe** (pink), ***αβαβ*‐UrFe** (green), ***αβ*‐UrFe** (cyan), ***αα*‐UrFe** (blue), **TPPFe** (black), **F_8_Fe** (dark gray), **F_20_Fe** (gray), under the same experimental conditions, and different atropisomers (***αααα***, ***α_3_β***, ***α_2_β_2_***, ***αβαβ***) of trimethylanilinium‐modified iron porphyrins (**FeTMA**) in DMF with 3 M PhOH as proton source (squares). (d) Plot of log TOF *vs* p*K_a_* of external acid for the different **UrFe** atropisomers. (e) Kinetic isotope effect (KIE) when switching from H_2_O to D_2_O during CO2RR. (f) Plot of log TOF *vs* concentration of water for the different **UrFe** atropisomers.

In our prior study on an iron porphyrin bearing two face‐to‐face urea arms (***αα*‐UrFe**), we showed protonation is rate‐determining [19, 26]. Kinetic isotope effect (KIE) measurements and DFT calculations revealed hindered proton (water) access to the active site by the urea arms. This interpretation was supported by a marked increase in the reaction rate upon removal of one urea arm in ***αβ*‐UrFe**, albeit at the expense of a reduced CO_2_ binding affinity. For ***α_2_β_2_*‐UrFe**, the second urea arm is repositioned adjacently rather than removed. As shown by the calculated buried volume (Figure 3f), this modification improves accessibility to the active site, and could also increase the local proton/water concentration by interaction with the partially bound urea moiety (Figure S37). This synergy is evidenced by pronounced upward trend in the log TOF with water (carbonic acid) as external proton source (Figure 4d). An exceptionally high KIE of 32 suggest that protons are involved in the rate determining steps (Figure 4e), consistent with the second‐order dependence on water concentration (Figure 5f), and distinct from the ***αβαβ*‐, *αα*‐,** and ***αβ*‐UrFe** atropisomers. This configuration results in a significantly enhanced TOFs (7.78 × 10^6^ s^−1^) at just 500 mV overpotential. This value can even reach 2.72 × 10^7^ s^−1^ at 7.22 M water, although some catalyst precipitation can be observed at such high‐water content. These results underscore once again the critical role of SCS topology and symmetry in fine‐tuning catalytic efficiency.

On the basis of the results described above, ***α_2_β_2_*‐UrFe** triggers the CO_2_‐to‐CO reduction from the Fe(I) species, identified as the catalytically active state to form the [Fe^III^─CO_2_]ˉ adduct [27]. From this common intermediate, two distinct mechanisms operate, depending on the reaction conditions (Scheme 3). Under protic conditions, it follows the conventional two‐electron/two‐proton pathway. Sequential or coupled proton and electron transfer generates [Fe^II^─CO_2_H]ˉ, whose further protonation cleaves the C─O bond (releasing H_2_O), followed by one‐electron reduction of [Fe^II^─CO] to produce CO and regenerate Fe(I). In contrast, in aprotic conditions the unique SCS configuration of ***α_2_β_2_*‐UrFe** enables insertion of a second CO_2_ molecule after one‐electron reduction of [Fe^III^─CO_2_]ˉ, leading in our calculation to a [Fe^II^─(CO_2_)_2_]^2^ˉ intermediate. This process is accompanied by a rearrangement of the urea groups that facilitates C─O bond cleavage and carbonate departure toward a distal urea arm, as evidenced by IR spectroscopy.

**SCHEME 3:**
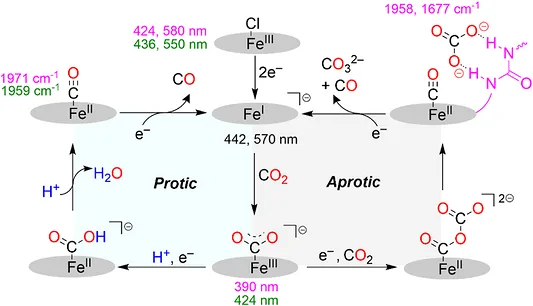
Proposed mechanistic divergence in protic (blue shade) *vs* aprotic (gray shade) CO2RR pathways for ***α_2_β_2_*‐UrFe** atropisomer, distinguished by UV–vis and IR signatures of intermediate species (***α_2_β_2_*** magenta, ***αβαβ*** *, green*).

## 3 Conclusion

We reported the first example of a modified iron porphyrin catalyst capable of promoting electrocatalytic CO_2_‐to‐CO reduction in the absence of both exogenous Brønsted and Lewis acids. This unprecedented reactivity arises from deliberate symmetry remodeling, which induces changes in directionality of the hydrogen‐bonding network within a urea‐based SCS. Our investigation demonstrates that positioning two urea arms within the same SCS at adjacent positions in ***α_2_β_2_*‐UrFe** fundamentally alters the interaction mode with the substrate coordinated to the metal center, compared to the face‐to‐face arrangement found in the previously studied ***αβαβ*‐UrFe** analogue. Mechanistic studies combining electrochemistry, spectroscopy, and DFT calculations reveal that, although the ***α_2_β_2_*** configuration provides comparatively weaker activation of the [Fe^III^─CO_2_]ˉ intermediate than the ***αβαβ*** arrangement, it uniquely enables insertion of a second CO_2_ molecule. This second CO_2_ acts as an oxygen‐atom acceptor, a process facilitated by reorganization of the urea arms and a more open steric environment (larger accessible buried volume) around the metal center. Under protic conditions, the enhanced accessibility of the active site in ***α_2_β_2_*‐UrFe** is also crucial, as it facilitates access of proton sources and thereby promotes proton transfer in the rate‐determining step. This effect contributes to a three‐order‐of‐magnitude increase in the reaction rate, reaching a TOF as high as 2.72 × 10^7^ s^−1^. More broadly, this study highlights an important aspect of secondary coordination sphere design: optimal catalysis not only result from the fine tune between stabilization and activation of key intermediates, but can also benefit from directional and adaptive non‐covalent interactions that can facilitate protonation and/or co‐substrate insertion. Beyond the simple proximity of functional groups to the active site, the preorganization and orientation of these interactions play a central role in controlling reactivity. In enzymes, such features are intrinsically imposed by the protein scaffold and likely contribute to the remarkable efficiency of metalloenzymes. In addition to recent developments in hybrid materials capable of achieving current densities more compatible with industrial applications [35, 36, 37, 38, 39], our approach opens up new perspectives for the design of efficient, enzyme‐inspired catalytic systems.

## Author Contributions

**Ashutosh Vishwakarma**: data curation, writing – review and editing, investigation, formal analysis. **Sami Essid**: investigation, formal analysis, data curation, writing – review and editing. **Sarah Bouery**: data curation, investigation. **Régis Guillot**: data curation, validation, formal analysis, investigation, writing – review and editing. **Bernard Boitrel**: conceptualization, formal analysis, writing – review and editing, investigation. **Philipp Gotico**: conceptualization, data curation, investigation, validation, formal analysis, supervision, writing – original draft. **Marie Sircoglou**: conceptualization, methodology, data curation, investigation, validation, formal analysis, supervision, writing – original draft. **Zakaria Halime**: conceptualization, methodology, investigation, validation, formal analysis, supervision, funding acquisition, project administration, visualization, resources, writing – original draft.

## Conflicts of Interest

The authors declare no conflicts of interest.

## Supporting information




**Supporting File**: The authors have cited additional references within the Supporting Information [40, 41, 42, 43, 44, 45, 46, 47, 48, 49, 50, 51, 52, 53, 54, 55, 56, 57, 58, 59, 60, 61, 62, 63, 64, 65, 66, 67, 68, 69, 70, 71, 72, 73, 74, 75, 76, 77, 78, 79, 80, 81, 82, 83].

## Data Availability

As indicated in the Supporting Information, the computational data that support the findings of this study will be openly available after the final acceptance of the article in figshare at: https://doi.org/10.6084/m9.figshare.31869178. For the reviewing process, reviewers can access the computational data if needed at this temporary link: https://figshare.com/s/6cdeaf1bb9978ceb570b.
