# Supplementary material for: Directional Second Sphere Effect on CO2 Activation and Reduction by Iron Porphyrin in Aprotic and Protic Media
Source: Angew Chem Int Ed Engl. 2026 Jul 23;65(42):e1490828. doi: 10.1002/anie.1490828 (PMC13653621; doi:10.1002/anie.1490828)
Supplement: Supplementary file 1 [file ANIE-65-e1490828-s001.docx]

Supporting Information

Directional Second Sphere Effect on CO_2_ Activation and Reduction by Iron Porphyrin in Aprotic and Protic Media

Ashutosh Vishwakarma,^+[a]^ Sami Essid,^+[a]^ Sarah Bouery,^[a]^ Régis Guillot,^[a]^ Bernard Boitrel,^[b]^ Philipp Gotico,*^[c]^ Marie Sircoglou*^[a]^ and Zakaria Halime*^[a]^

[a]	A. Vishwakarma, S. Essid, S. Bouery, Dr. R. Guillot, Dr. M. Sircoglou, Dr. Z. Halime
Université Paris-Saclay, CNRS, Institut de Chimie Moléculaire et des Matériaux d'Orsay, 91400, Orsay, France.
E-mail: marie.sircoglou@universite-paris-saclay.fr, zakaria.halime@universite-paris-saclay.fr

[b]	Dr. B. Boitrel
Univ Rennes, CNRS, ISCR (Institut des Sciences Chimiques de Rennes)-UMR 6226, 35000, Rennes, France.

[c]	Dr. P. Gotico
Institute for Integrative Biology of the Cell, CEA, CNRS, Université Paris-Saclay, 91191, Gif-sur-Yvette, France. E-mail: philipp.gotico@cea.fr

[+]	These authors contributed equally to this work.

Table of Contents

[Table of Contents	2](#_Toc231495665)

[Experimental Procedures	3](#_Toc231495666)

[A. General Materials and Methods	3](#_Toc231495667)

[B. Synthesis and Characterization of Complexes	3](#_Toc231495668)

[C. Electrochemical Characterization	5](#_Toc231495669)

[Results and Discussion	6](#_Toc231495670)

[A. Electrochemical Characterization	6](#_Toc231495671)

[B. Controlled Potential Electrolysis (CPE)	12](#_Toc231495672)

[C. Kinetic Isotope Effect (KIE)	13](#_Toc231495673)

[D. UV-Visible Spectroelectrochemistry (UV-Vis SEC)	14](#_Toc231495674)

[E. FTIR Spectroelectrochemistry (FTIR SEC)	16](#_Toc231495675)

[F. X-Ray Diffraction Crystallography	17](#_Toc231495676)

[G. UV-Visible, Nuclear Magnetic Resonance, and Mass Spectra	20](#_Toc231495677)

[H. Computational Details	26](#_Toc231495678)

[References	33](#_Toc231495679)

Experimental Procedures

A. General Materials and Methods

Dimethylformamide (DMF, Aldrich 99.9%), tetra-*N*-butylammonium hexafluorophosphate ([Bu_4_N]PF_6_, Aldrich 99%) were used as received. All other chemical reagents used in the synthetic route were obtained from commercial sources as guaranteed-grade reagents and used without further purification. Water was Milli-Q filtered (18.2 MΩ·cm at 25 °C).

X-ray diffraction data for compound ***α_2_β_2_*-UrFe** were collected by using a VENTURE PHOTONIII c7 Bruker diffractometer with Micro-focus IuS source Mo-K*α* radiation. Crystals of compounds were selected under optical microscope and glued in paratone oil. Crystal was mounted on a CryoLoop (Hampton Research) with Paratone-N (Hampton Research) as cryoprotectant and then placed in a nitrogen-gas stream at low temperature. The temperature of the crystal was maintained at the selected value by means of a cooling device by a 700 series Cryostream cooling device to with an accuracy of ±1K. Data reduction was accomplished using SAINT V7.53a. The substantial redundancy in data allowed a semi-empirical absorption correction (SADABS V2.10) to be applied, on the basis of multiple measurements of equivalent reﬂections. The structures were solved by direct methods using SHELXS-97^[1]^ and refined against *F*^2^ by full-matrix least-squares techniques using SHELXL-2025^[2]^ with anisotropic displacement parameters for all non-hydrogen atoms. All calculations were performed by using the Crystal Structure crystallographic software package WINGX.^[3]^ These data can be obtained free of charge from the Cambridge Crystallographic Data Centre and Fachinformationszentrum Karlsruhe via http://www.ccdc.cam.ac.uk/structures/.

Proton and carbon nuclear magnetic resonance (^1^H and ^13^C NMR) spectra were recorded at room temperature on Bruker Advance 300 MHz spectrometers. Two-dimensional NMR (COSY, HSQC and HMBC) experiments were also performed in order to confirm the attribution of one-dimensional ^1^H NMR signals. The electrospray ionization mass spectrometry (ESI-HRMS) experiments were performed on TSQ (Thermo Scientific. 2009) with an ESI^+^ method. Ground state absorption spectra were measured on a Specord double beam spectrophotometer.

B. Synthesis and Characterization of Complexes

**Figure S1.** Synthetic scheme for the preparation of ***α_2_β_2_*-UrFe**

| **Synthesis of porphyrin *α_2_β_2_*-Ur:**  3,5-Bis(trifluoromethyl)phenyl isocyanate (165 µL, 951 µmol) were added to a solution of ***α_2_β_2_*-amino** (100 mg, 148 µmol) in dry dichloromethane (10 mL) under argon. After 3h stirring at room temperature, the solvent was removed under vacuum. The expected compound was precipitated by dissolving the reaction crude it in a minimum amount of dichloromethane and adding a large amount of hexane. After a filtration, washing with pentane and drying under vacuum, porphyrin ***α_2_β_2_*-Ur** was obtained as solid with 88 % yield (221 mg). | |
| --- | --- |
| **^1^H NMR:** ((CD_3_)_2_CO, 298 K, 300 MHz): δ_H_ 8.92(4H, s, *β*_pyrrole_), 8.90(4H, s, *β*_pyrrole_) 8.47 (4H, d, H_arom_), 8.0 (4H, s, H_arom_), 7.90 (8H, m, H_arom,_ NH_urea_), 7.56 (4H, t, H_arom_), 7.53 (8H, s, H_arom_), 7.41 (4H, s, NH_urea_), 7.30(4H, s, H_arom_), -2.70 (2H, s, NH_pyrrole_).  **^13^C NMR:** ((CD_3_)_2_CO, 298 K, 75 MHz): δ_c_ 153.97, 153.90, 142.81, 140.57, 136.83, 134.31, 133.29, 132.96, 132.63, 132.30, 132.28, 128.85, 126.15, 124.71, 124.58, 123.45, 119.26, 117.26, 117.26, 116.15.  **ESI-HRMS:** calculated m/z = 1717.32739 [M+Na^+^]^+^ for C_80_H_46_F_24_N_12_O_4_, found 1717.32791.  **UV-Vis absorption peaks in acetone, λ in nm (ε in M^-1^cm^-1^):** 421(359000), 515(21000), 548(5000), 591(7000), 645(2000). |  |
| **Synthesis of iron porphyrin *α_2_β_2_*-UrFe:**  A solution of porphyrin ***α_2_β_2_*-Ur** (206 mg, 122 µmol) and lutidine (424 µL, 3.66 mmol) in dry THF was degassed by argon bubbling for 30 min. Then, FeBr_2_ (181 mg, 0.84 mmol) was added and the reaction mixture was stirred overnight at room temperature under argon. The reaction mixture was then filtered to remove the excess of iron salts and the solvent was evaporated under vacuum. The reaction crude was dissolved in 100 mL of CHCl_3_ and 100 mL aqueous solution of 1 M HCl was added. After, 3 hours stirring the two phases were separated and the organic phase containing the iron complex was evaporated under vacuum. Chromatographic purification on a silica gel using a CH_2_Cl_2_ as eluent gives ***α_2_β_2_*-UrFe** (with a Cl^-^ as axial coordinating anion) in 92 % yield (200 mg).  **ESI-HRMS:** calculated m/z = 1802.26536 [M-Cl^-^ + CH_3_ONa]^+^ for C_81_H_47_F_24_FeN_12_O_5_, found 1802.26574.  **UV-Vis absorption peaks in DMF, λ in nm (ε in M^-1^cm^-1^):** 424 (177000), 580 (32000). |  |

C. Electrochemical Characterization

**Cyclic Voltammetry**

Cyclic voltammetry measurements were performed using a potentiostat (CH Instruments or Metrohm Autolab PGSTAT128N) in a three-electrode electrochemical cell consisting of a glassy carbon working electrode (3 mm diameter), a standard calomel electrode (SCE) as the reference electrode, and a platinum wire counter electrode. Before each scan, the electrode was carefully polished with diamond paste (1 *µ*m), sonicated with ethanol, washed with ethanol and acetone, and dried. Ohmic drop was compensated for each experiment. Dimethylformamide (DMF, 99.8%, Extra Dry, AcroSeal, Thermo Scientific Chemicals) was mainly used as solvent. The solvent was transferred under a dry argon atmosphere using Schlenk line techniques, and the electrochemical cells were systematically purged with dry argon or CO_2_. Solutions of samples were prepared at a concentration of 1 mM. Tertbutylammonium hexafluorophosphate ([Bu_4_N]PF_6_) was used as supporting electrolyte and its concentration was maintained at hundred-fold excess compared to the sample. Different proton sources (water, trifluoroethanol, phenol) were added, the solutions were purged with argon or CO_2_ gas and the cyclic voltammograms were measured at a scan rate of 100 mV/s (unless stated otherwise). For better comparison with literature data, potentials were converted to the ferrocene/ferrocenium (Fc/Fc^+^) reference.

The concentration of CO_2_ in the solution was controlled using two gas flow meters purchased from Bronkhorst and adjusting the CO_2_ to argon partial pressure. The gases were mixed properly in a gas mixer before purging into the electrolyte solution for 15 minutes. During CV measurements, the same gas ratio was maintained in the headspace of the cell.

**Controlled Potential Electrolysis (CPE) and Product Analysis**

CPE experiments were undertaken using controlled potential for 2 hours with a Metrohm Autolab PGSTAT128N potentiostat, in a H-cell system with one compartment for the anolyte (12 mL) with the counter electrode, and the other compartment for the catholyte (6 mL) with the working electrode and the reference electrode (in a tube containing the electrolyte, separated with a frit); Bu_4_NPF_6_ (0.1 M) in DMF: H_2_O (10:1) was chosen as electrolyte. The catalyst was dissolved in 6 mL of electrolyte at 0.5 mM concentration. Both electrolytes were bubbled with CO_2_ for 20 min, the cell was sealed with rubber septa and the tightness was checked using the *µ*GC. A glassy carbon rod was used as working electrode, platinum mesh was used as counter electrode and a SCE electrode was used as reference. The conversion from SCE to Fc/Fc^+^ was done by performing CV of Fc/Fc^+^ using the H-cell setup.

**Figure S2.** Sketch of the H-cell setup used for CPE.

Gas products were detected by gas chromatography (Micro GC Fusion Gas Analyser, Inficon) with two modules having Ar carrier gas with 99.999% purity and TCD detectors. In module A (PLOT column with molecular sieve and 10 m length) a 9 s backflush is applied to separate H_2_, O_2_, N_2_, CH_4_ and CO. In Module B (RT-Q-bond column of 12 m) allows the detection of heavier molecules, such as CO_2_, H_2_O vapor and C_1_/C_2_ products. Relevant to the experiments, the following gases were detected in Module A at specific retention times: H_2_ (44.54 s), O_2_ (53.54 s), N_2_ (49.56 s), CO (59.34 s). The amounts of H_2_ and CO were calibrated using the same electrochemical setup for CPE, with the same volume of electrolytes (Figure S2).

|  |  |
| --- | --- |
| **Figure S3.** Calibration curves of the H-cell used for CPE experiments: CO (left), H_2_ (right). | |

**FTIR Spectroelectrochemistry (FTIR-SEC)**

The FTIR data were collected on the Bruker Vertex 80 instrument. The optically transparent thin-layer electrochemical (OTTLE) cell for spectroelectrochemistry was used. Deuterated acetonitrile (d_3_-ACN) solutions of the ***α_2_β_2_*-UrFe**, and ***αβαβ*-UrFe** (300 μL; 1 mM) were prepared and purged with CO_2_ for 10 min. The sample vial was sealed properly and re-inserted. The samples were injected in the OTTLE cell (200 μL) and tightly sealed. Spectroelectrochemistry was performed using the OTTLE cell connected with the potentiostat (CH Instruments). The sample solution contained 100 mM [Bu_4_N]PF_6_ as the supporting electrolyte. Pt-mesh electrodes were used both as working and counter electrodes and silver wire was used as the reference electrode. The potential of the reference electrode was calibrated using standard ferrocene solution (1 mM) in the same solvent combination. The FTIR spectra were recorded at different time intervals under electrolysis conditions at different potentials.

**UV-Visible Spectroeclectrochemistry (UV-Vis SEC)**

UV-Vis SEC was performed on an Agilent Cary 60 spectrophotometer using a Squidstat potentiostat (Admiral Instruments), with the spectral window set from 300 nm to 800 nm. Solutions of the ***α_2_β_2_*-UrFe**, and ***αβαβ*-UrFe** (50 μM) containing 100 mM [Bu₄N]PF₆ as supporting electrolyte were prepared. Measurements were carried out using the Honeycomb Spectroelectrochemical Cell Kit (AKSTCKIT3), which includes a 1 mm path-length quartz cuvette, cell cap, a platinum honeycomb electrode, and the Universal Specialty Cell Connection Kit, with the patterned honeycomb electrode chip incorporating on board working, counter, and reference electrodes. UV-Vis spectra were collected at defined time intervals during electrolysis at various applied potentials.

Results and Discussion

A. Electrochemical Characterization

**Table S1.** Formal reduction potentials (*vs* Fc/Fc^+^) of the studied complexes (1 mM concentration) in Ar-purged (or CO-purged) DMF containing and 0.1 M [Bu_4_N]PF_6_.

| **Complexes** | **Fe^III/II^** | **Fe^II/I^** | **Fe^I/0^** | **Fe^II/I^-CO** |
| --- | --- | --- | --- | --- |
| ***α_2_β_2_*-UrFe** | -0.49 | -1.37 | -1.89 | -1.69 |
| ***αβαβ*-UrFe** | -0.45 | -1.29 | -1.80 | -1.53 |
| ***αβ*-UrFe**^[4]^ | -0.52 | -1.38 | -1.98 | not measured |
| ***αα*-UrFe**^[4]^ | -0.52 | -1.37 | -1.93 | not measured |

**Foot-of-the-Wave Analysis**^[5,6]^

For fast catalytic process, the foot-of-the-wave (FOW) analysis of the CVs has been performed as a quick estimation of the catalytic rate constant, *k_cat_* and TOF of the catalytic reaction without contribution from side phenomena such as substrate consumption, catalyst deactivation, and/or product inhibition. The analysis is based on the linear correlation between *i/i_p_^0^* and *1/{1+exp[F/RT(E - E^0^_cat_)]}* where:

*i*: catalytic current in the presence of CO_2_ and proton source (corrected with the background capacitive current, *i_c_, see a step-by-step method reported by Kubiak et.al.*^[7]^)

*i_p_^0^*: peak current in the absence of CO_2_ which was normalized to the reversible redox couple Fe^II/I^ under argon

*F*: faraday constant

*R*: gas constant

*T*: absolute temperature

Plotting *i/i_p_^0^ vs. 1/{1+exp[F/RT(E - E^0^_cat_)]}* gives rise to a straight line (Figures S3 – S6, S8, S14b) with a slope = *2.24(RT/Fν)^1/2^(2k_cat_)^1/2^* where *ν* is the scan rate. It is important to note that the linear fits (R^2^ > 0.98) in the initial range (foot-of-the wave) should include *i/i_p_^0^* > 1 (catalytic condition) while maintaining the ideal y-intercept of 0, as similarly considered by Dey et. al.^[8]^

$$\frac{i}{i_{p}^{0}}=\frac{2.24\sqrt{\frac{RT}{Fv}{2k}_{cat}}}{1+exp\left[ \frac{F}{RT}\left( E-E_{cat}^{0} \right) \right]}$$

The corresponding *k_cat_* can be calculated form this slope and the catalytic Tafel plot (log TOF *vs* $\eta$) can be constructed:

$$TOF=\frac{k_{cat}}{1+exp\left[ \frac{F}{RT}\left( E-E_{cat}^{0} \right) \right]}$$

$$For reduction reactions, \eta=E_{{{CO}_{2}}/{CO}}^{0}-E$$

$E_{{{CO}_{2}}/{CO}}^{0}=-1.38 V vs Fc in DMF:H_{2}O$ (for derivation, see SI of a study by Savéant et. al$ADDIN ZOTERO\_ITEM CSL\_CITATION \{"citationID":"mbE7lrtE","properties":\{"formattedCitation":"\backslash\backslash super [9]\backslash\backslash nosupersub\{\}","plainCitation":"[9]","noteIndex":0\},"citationItems":[\{"id":"Cq0EUu9n/qVhQmogs","uris":["http://zotero.org/users/2541287/items/M8FPYJB2",["http://zotero.org/users/2541287/items/M8FPYJB2"]],"itemData":\{"id":370,"type":"article-journal","abstract":"Redox pairs of transition metal complexes are often involved in small molecule activation in response to modern energy challenges as well as in other areas of electrocatalysis. Within such a family of molecular electrocatalysts, ligand substitution is a means of varying catalytic efficiency, best gauged through catalytic Tafel plots relating overpotential and turnover frequency. In practice, efficient molecular catalysis involves multielectron-multistep processes. It is in this framework that we discuss through-structure inductive substituent effects. What the best choice is for the reference thermodynamic index, how the global substituent effect may be expressed as a function of this index, and how it may be dissected into individual effects assigned to each of the reaction steps are challenging questions that are addressed and resolved here for the first time. The discussion is illustrated by the effect of successive phenyl perfluoration and of o,o'-methoxy substitution of the FeI/0 tetraphenylporphyrin catalysts of the CO2-to-CO electrochemical conversion. Consequences on the relative position of the catalytic Tafel plots are also examined. This analysis of through-structure electronic effects is a necessary preliminary to the investigation of substituent through-space effects (electrostatic, H-bonding) because, albeit of different nature, they may occur simultaneously. Investigation of these two aspects of substituent effects and of the rules that emerge thereof pave the way to future imaginative design of catalysts for the CO2-to-CO-conversion and also for any other molecular catalytic reactions.","container-title":"The Journal of Physical Chemistry C","DOI":"10.1021/acs.jpcc.6b09947","ISSN":"1932-7447","issue":"51","journalAbbreviation":"J. Phys. Chem. C","page":"28951-28960","source":"ACS Publications","title":"Dissection of Electronic Substituent Effects in Multielectron-Multistep Molecular Catalysis. Electrochemical CO2-to-CO Conversion Catalyzed by Iron Porphyrins","volume":"120","author":[\{"family":"Azcarate","given":"Iban"\},\{"family":"Costentin","given":"Cyrille"\},\{"family":"Robert","given":"Marc"\},\{"family":"Savéant","given":"Jean-Michel"\}],"issued":\{"date-parts":[["2016",12,29]]\}\}\}],"schema":"https://github.com/citation-style-language/schema/raw/master/csl-citation.json"\}$^[9]^)

$$TOF=\frac{k_{cat}}{1+exp\left[ \frac{F}{RT}\left( E_{{{CO}_{2}}/{CO}}^{0}-E_{cat}^{0}- \eta\right) \right]}$$

$$logTOF=logk_{cat}- \frac{F}{RTln10}(E_{{{CO}_{2}}/{CO}}^{0}-E_{cat}^{0})+\frac{F}{RTln10}\eta$$

$$\mathrm{If}\eta=0, E_{{{CO}_{2}}/{CO}}^{0}=E, then$$

$${logTOF}_{0}=logk_{cat}- \frac{F}{RTln10}(E_{{{CO}_{2}}/{CO}}^{0}-E_{cat}^{0})$$

$$At low values of \eta\left( E_{{{CO}_{2}}/{CO}}^{0}>E> E_{cat}^{0} \right): logTOF={logTOF}_{0}+\frac{F}{RTln10}\eta$$

$$At high values of \eta\left( E< E_{cat}^{0} \right): logTOF={logTOF}_{max}={logk}_{cat}$$

|  |  |
| --- | --- |
| **Figure S4.** Cyclic voltammograms (left) and FOW analysis (right) of 1 mM ***α_2_β_2_*-UrFe** in Ar-saturated (black) and CO_2_-saturated (red) DMF with varying concentrations of H_2_O (light to dark blue: 0.55, 1.11, 1.66, 2.22, 2.77, 3.33, 3.88, 4.44, 5.00, 5.55, 6.11, 6.66 M) showing linear fits used to determine *k*_cat_. The oxidative scan is omitted for clarity. | |
|  |  |
| **Figure S5.** Cyclic voltammograms (left) and FOW analysis (right) of 1 mM ***α_2_β_2_*-UrFe** in Ar-saturated (black) and CO_2_-saturated (red) DMF with varying concentrations of TFE (light to dark brown: 0.55, 1.11, 1.66 M) showing linear fits used to determine *k*_cat_. The oxidative scan is omitted for clarity. | |
|  |  |
| **Figure S6.** Cyclic voltammograms (left) and FOW analysis (right) of 1 mM ***α_2_β_2_*-UrFe** in Ar-saturated (black) and CO_2_-saturated (red) DMF with varying concentrations of PhOH (light to dark blue: 0.55, 1.11 M) showing linear fits used to determine *k*_cat_. The oxidative scan is omitted for clarity. | |

|  |  |
| --- | --- |
| **Figure S7.** Cyclic voltammograms (left) and FOW analysis (right) of 1 mM ***αβαβ*-UrFe** in Ar-saturated (black) and CO_2_-saturated (red) DMF with varying concentrations of H_2_O (light to dark blue: 0.55, 1.11, 1.66, 2.22, 2.77, 3.33, 3.88, 4.44, 5.00, 5.55 M) showing linear fits used to determine *k*_cat_. The oxidative scan is omitted for clarity. | |

|  |  | |  | |
| --- | --- | --- | --- | --- |
| **Figure S8.** Comparison of cyclic voltammograms of ***α_2_β_2_*-UrFe** (magenta) and ***αβαβ*-UrFe** (green) in Ar-saturated (left), CO_2_-saturated (center) and with 5.55 M H_2_O (right) in DMF. | | | | |
|  | |  | |  |
| **Figure S9.** Cyclic voltammograms of 0.5 mM ***α_2_β_2_*-UrFe** (left) in Ar-saturated and with varying concentrations of CO_2_ (light to dark pink/green: 0.046, 0.092, 0.138, 0.184, 0.230 M) in DMF and FOW analysis (rightr) showing linear fits used to determine *k*_cat_. The oxidative scan is omitted for clarity. | | | | |

|  |  | |
| --- | --- | --- |
| **Figure S10.** Cyclic voltammograms of 0.5mM ***α_2_β_2_*-UrFe** (left) and ***αβαβ*-UrFe** (center) in Ar- (black) and CO-saturated (red) DMF. | |  |

|  |  | |
| --- | --- | --- |
| **Figure S11.** Differential pulse voltammograms of 0.5mM ***α_2_β_2_*-UrFe** (left) and ***αβαβ*-UrFe** (center) in Ar- (black) and CO-saturated (red) DMF. | |  |

**Table S2.** Calculated catalytic rate constants from the foot-of-the-wave analysis of the cyclic voltammograms of the complexes investigated at various conditions.

| **Complex** | **Proton Source** | **Conc / M** | ***k*_cat_ / s^-1^** | **log (*k*_cat_ / s^-1^)** |  |
| --- | --- | --- | --- | --- | --- |
| ***α_2_β_2_*-UrFe** | CO_2_ | 0.046 | 30.4 | 1.48 |  |
|  | CO_2_ | 0.092 | 104 | 2.02 |  |
|  | CO_2_ | 0.138 | 306 | 2.48 |  |
|  | CO_2_ | 0.184 | 682 | 2.83 |  |
|  | CO_2_ | 0.230 | 1300 | 3.12 |  |
|  | H_2_O | 0.55 | 23800 | 4.38 |  |
|  | H_2_O | 1.11 | 152000 | 5.18 |  |
|  | H_2_O | 1.66 | 232000 | 5.36 |  |
|  | H_2_O | 2.22 | 458000 | 5.66 |  |
|  | H_2_O | 2.77 | 811000 | 5.91 |  |
|  | H_2_O | 3.33 | 1610000 | 6.21 |  |
|  | H_2_O | 3.88 | 2180000 | 6.34 |  |
|  | H_2_O | 4.44 | 3800000 | 6.57 |  |
|  | H_2_O | 5.00 | 5000000 | 6.70 |  |
|  | H_2_O | 5.55 | 7780000 | 6.89 |  |
|  | H_2_O | 6.11 | 12200000 | 7.09 |  |
|  | H_2_O | 6.66 | 19600000 | 7.29 |  |
|  | H_2_O | 7.22 | 27200000 | 7.43 |  |
|  | TFE | 0.55 | 14700 | 4.17 |  |
|  | TFE | 1.11 | 23300 | 4.36 |  |
|  | TFE | 1.66 | 124000 | 5.09 |  |
|  | PhOH | 0.55 | 546000 | 5.73 |  |
|  | PhOH | 1.11 | 710000 | 5.85 |  |
| ***αβαβ*-UrFe** | H_2_O | 0.55 | 105 | 2.02 |  |
|  | H_2_O | 1.11 | 301 | 2.49 |  |
|  | H_2_O | 1.66 | 349 | 2.54 |  |
|  | H_2_O | 2.22 | 596 | 2.77 |  |
|  | H_2_O | 2.77 | 1020 | 3.01 |  |
|  | H_2_O | 3.33 | 1730 | 3.24 |  |
|  | H_2_O | 3.88 | 2960 | 3.47 |  |
|  | H_2_O | 4.44 | 3777 | 3.58 |  |
|  | H_2_O | 5.00 | 4640 | 3.67 |  |
|  | H_2_O | 5.55 | 5680 | 3.75 |  |
|  | | | | | |
| **Figure S12.** Comparison of catalytic Tafel plots [log(TOF) *vs* overpotential (*η*)] calculated using FOW analysis of ***α_2_β_2_*-UrFe** (magenta), ***αβαβ*-UrFe** (green), **TPPFe** (black), **F_8_Fe** (red), and **F_20_Fe** (blue) under the same experimental conditions. Condition: CO_2_-purged DMF containing 5.55 M H_2_O and 0.1 M [Bu_4_N]PF_6_. | | | | | |

**Diffusion Coefficient**

The diffusion coefficients of the catalysts were determined by plotting peak current as a function of square root of scan rate, following Randles-Sevcik equation:

$$i_{p}=0.4463nFAC\left( \frac{nFvD}{RT} \right)^{\frac{1}{2}}$$

**Figure S13.** Diffusion current peaks (Fe^II/I^: reduction in blue, oxidation in orange) of 1 mM ***α_2_β_2_*-UrFe** in Ar-saturated DMF containing 0.1M [Bu_4_N]PF_6_, as a function of square root of scan rate.

The following parameters were used: *n* (electron transferred) = 1, *F* = 96485 C mol^-1^, *R* = 8.314 J K^-1^ mol^-1^, *T* = 298 K, *A* = 0.07065 cm^2^ (glassy carbon electrode with diameter of 3 mm), *C* = 1 × 10^-6^ mol cm^–3^. The calculated average diffusion coefficient of ***α_2_β_2_*-UrFe** is 6.92 × 10^-7^ cm^2^ s^-1^.

**Table S3.** Calculated diffusion coefficients of the complexes in DMF.

| **Complexes** | D / × 10^-6^cm^2^ s^-1^ |
| --- | --- |
| ***α_2_β_2_*-UrFe** | 0.69 |
| ***αβαβ*-UrFe**^[10]^ | 1.40 |
| ***αβ*-UrFe**^[4]^ | 1.12 |
| ***αα*-UrFe**^[4]^ | 0.89 |

B. Controlled Potential Electrolysis (CPE)

Faradic efficiency (FE) is determined from the *Q_exp_* / *Q_theo_* for the 2-electron reduction of CO_2_ to CO. *Q_theo_* is the total charge passed taken from charge *vs* time curves (Figure S14) while *Q_exp_* is the total charge derived from the amount of CO detected by GC (*Q_exp_* = 2 × F × mol_CO_ ).

|  |  |
| --- | --- |
|  |  |
| **Figure S14.** Plot of current density (red) and charge (blue) as a function of time for 0.5 mM ***α_2_β_2_*-UrFe** in a CO2-saturated DMF containing 0.1 M [Bu4N]PF6 in the absence (top) and presence (bottom) of 5.55 M H2O during bulk electrolysis (*E*electrolysis = -1.86 V *vs* Fc/Fc^+^). | |

The electrolysis is described by the equation^[5,11]^:

$$\frac{i}{FA}=\frac{C_{cat}^{0}\sqrt{2k_{cat}D_{cat}}}{1+\exp\left[ \frac{F}{RT}\left( E_{electrolysis}-E_{cat} \right) \right]}$$

$$k_{cat}=\frac{i^{2}\left[ 1+\exp\left[ \frac{F}{RT}\left( E_{electrolysis}-E_{cat} \right) \right] \right]^{2}}{2F^{2}A^{2}{C_{cat}^{0}}^{2}D_{cat}}$$

**Table S4.** Catalyst performances for ***α_2_β_2_*-UrFe** calculated from CPE in CO_2_-saturated DMF solution without and with 5.55 M H_2_O after 2 hours.

| **Conditions** | **Q_exp_(CO)** | **Q_exp_(H_2_)** | **Q_theo_** | **FE (CO)** | **FE (H_2_)** |
| --- | --- | --- | --- | --- | --- |
| **Without H_2_O** | 0.18 | 0.00 | 0.62 | 29% | 0% |
| **With 5.55 M H_2_O** | 6.45 | 0.15 | 7.70 | 84% | 2% |

**For CPE without H_2_O:**

Calculations were made from *i* = -0.03 mA (taking into account 29% FE for CO production) and the following parameters: *A* (electrode surface) = 0.6597 cm^2^, *C^0^_cat_* (catalyst concentration) = 5 x 10^-7^ mol cm^-3^, *D_cat_* (catalyst diffusion coefficient) = 6.92 × 10^-7^ cm^2^ s^-1^, *E*_electrolysis_ = -1.86 V *vs.* Fc/Fc^+^, *E*_cat_ = -1.89 V *vs.* Fc/Fc^+^ *F* = 96 485 C mol^-1^, *R* = 8.314 J K^-1^ mol^-1^, *T* = 298 K, and *t* (electrolysis time) = 7200 s. The calculated value for the catalytic rate constant k_cat_ is 13.8 s^-1^.

The turnover frequency (TOF) and turnover number (TON) was then calculated:

$$TOF= \frac{k_{cat}}{1+\exp\left[ \frac{F}{RT}\left( E_{electrolysis}-E_{cat} \right) \right]}=0.28 s^{-1}$$

$$TON=TOF\times t=2016 (based on catalyst conc. on diffusion layer)$$

$$TON'= \frac{mol of CO after 2 hr electrolysis}{mol of cat} =0.31 (based on bulk conc. of catalyst)$$

**For CPE with 5.55 M H_2_O:**

Calculations were made from *i* = -1.15 mA (taking into account 84% FE for CO production) and the following parameters: *A* (electrode surface) = 0.6597 cm^2^, *C^0^_cat_* (catalyst concentration) = 5 x 10^-7^ mol cm^-3^, *D_cat_* (catalyst diffusion coefficient) = 6.92 × 10^-7^ cm^2^ s^-1^, *E*_electrolysis_ = -1.86 V *vs.* Fc/Fc^+^, *E*_cat_ = -1.89 V *vs.* Fc/Fc^+^ *F* = 96485 C mol^-1^, *R* = 8.314 J K^-1^ mol^-1^, *T* = 298 K, and *t* (electrolysis time) = 7200 s. The calculated value for the catalytic rate constant k_cat_ is 7.33 × 10^3^ s^-1^.

The turnover frequency (TOF) and turnover number (TON) was then calculated:

$$TOF= \frac{k_{cat}}{1+\exp\left[ \frac{F}{RT}\left( E_{electrolysis}-E_{cat} \right) \right]}=2827 s^{-1}$$

$$TON=TOF\times t=2.03\times{10}^{7} (based on catalyst conc. on diffusion layer)$$

$$TON'= \frac{mol of CO after 2 hr electrolysis}{mol of cat} =11 (based on bulk conc. of catalyst)$$

C. Kinetic Isotope Effect (KIE)

|  |  |
| --- | --- |
| **Figure S15a.** Comparison of cyclic voltammograms of 1 mM ***α_2_β_2_*-UrFe** in Ar-saturated (black) and CO_2_-saturated DMF containing 3.88 M H_2_O (blue) or 3.88 M D_2_O (purple). Inset shows magnified FOW. The oxidative scan is omitted for clarity. | **Figure S15b.** FOW analysis of 1 mM ***α_2_β_2_*-UrFe** in CO_2_-saturated DMF as a function of D_2_O concentration (light to dark purple: 0.55, 1.11, 1.66, 2.22, 2.77, 3.33, 3.88 M), showing linear fits used to determine *k*_cat_. |

**Table S5.** Calculated catalytic rate constants from the foot-of-the-wave analysis of the cyclic voltammograms of the complexes comparing the effect of H_2_O or D_2_O as proton source, with the corresponding calculated Kinetic Isotope Effect (KIE).

| **Complex** | **Conc / M** | ***k*_cat, H2O_ / s^-1^** | ***k*_cat, D2O_ / s^-1^** | **KIE (*k*_cat, H2O_ / *k*_cat, D2O_)** |
| --- | --- | --- | --- | --- |
| ***α_2_β_2_*-UrFe** | 0.55 | 23800 | 2280 | 10 |
|  | 1.11 | 152000 | 3720 | 41 |
|  | 1.66 | 232000 | 5570 | 42 |
|  | 2.22 | 458000 | 16200 | 28 |
|  | 2.77 | 811000 | 31900 | 25 |
|  | 3.33 | 1610000 | 60600 | 27 |
|  | 3.88 | 2180000 | 65700 | 33 |

D. UV-Visible Spectroelectrochemistry (UV-Vis SEC)

|  |  | |  | |
| --- | --- | --- | --- | --- |
| **Figure S16.** UV-Vis spectra of ***α_2_β_2_*-UrFe** in N_2_-saturated DMF showing the stepwise reduction of the iron center: Fe(III) to Fe(II) (black to red), Fe(II) to Fe(I) (red to blue), and Fe(I) to Fe(0) (blue to green), recorded at progressively more negative potentials. | | | | |
|  |  | |  | |
| **Figure S17.** UV-Vis spectra of ***αβαβ*-UrFe** in N_2_-saturated DMF showing the stepwise reduction of the iron center: Fe(III) to Fe(II) (black to red), Fe(II) to Fe(I) (red to blue), and Fe(I) to Fe(0) (blue to green), recorded at progressively more negative potentials. | | | | |
|  | |  | |  |
| **Figure S18.** UV-Vis spectra of ***α_2_β_2_*-UrFe** in CO_2_-saturated dry DMF showing the stepwise reduction of the iron center: Fe(III) to Fe(II) (black to red), and Fe(II) to Fe(I)-CO_2_ (red to brown), recorded at progressively more negative potentials. | | | |  |
|  | |  | |  |
| **Figure S19.** UV-Vis spectra of ***αβαβ*-UrFe** in CO_2_-saturated dry DMF showing the stepwise reduction of the iron center: Fe(III) to Fe(II) (black to red), and Fe(II) to Fe(I)-CO_2_ (red to brown), recorded at progressively more negative potentials. | | | |  |
|  | |  | |  |
| **Figure S20.** UV-Vis spectra of Fe(II) (red) and Fe(II)CO (purple) species for ***α_2_β_2_*-UrFe** (left) and ***αβαβ*-UrFe** (right) CO-saturated DMF. | | | |  |

E. FTIR Spectroelectrochemistry (FTIR SEC)

|  |  |
| --- | --- |

**Figure S21.** FTIR-SEC spectra 1mM of ***α_2_β_2_*-UrFe** (left) and ***αβαβ*-UrFe** (center) in anhydrous CO_2_-saturated deuterated acetonitrile with 0.1 M [Bu_4_N]PF_6_ supporting electrolyte, Ag-wire pseudo reference electrode, and Pt-mesh as both counter and working electrodes. The potentials are reported *vs* Fc^+/0^. The detection of the Fe(II)-CO species at early potentials is due to the presence of very limited traces of water, that we hypothesize to be immediately consumed upon applying further negative potentials, making the conditions more aprotic.

**Table S6.** Summary of electrochemical properties and spectroscopic data of ***α_2_β_2_*-UrFe and *αβαβ*-UrFe**.

| **Catalysts** | ***α_2_β_2_*-UrFe** | ***αβαβ*-FeUr** |
| --- | --- | --- |
| **E (Fe^II/I^) / V *vs* Fc/Fc^+^** | -1.37 | -1.29 |
| **E (Fe^II/I^-CO) / V *vs* Fc/Fc^+^** | -1.69 | -1.53 |
| **E (Fe^I/0^) / V *vs* Fc/Fc^+^** | -1.89 | -1.80 |
| **η / V** | 0.50 | 0.42 |
| **TOF_max_ / s^-1^** | 7780000 | 5680 |
| **log (TOF_max_)** | 6.89 | 3.75 |
| **KIE (k_cat,H2O_ /k_cat,D2O_)** | 31.9 | 5.77^[10]^ |
| **λ_max_ (Fe^III^) / nm** | 424, 580 | 436, 550 |
| **λ_max_ (Fe^II^) / nm** | 442, 532, 570, 614 | 442, 532, 570, 614 |
| **λ_max_ (Fe^I^) / nm** | 318, 394, 432, 512, 580 | 318, 394, 432, 512, 580 |
| **λ_max_ (Fe^0^) / nm** | 318, 360, 436, 462, 530 | 318, 360, 436, 458, 530 |
| **λ_max_ (Fe^I^-CO_2_) / nm** | 390, 442, 570 | 424, 442, 570 |
| **λ_max_ (Fe^II^-CO) / nm** | 426, 540 | 426, 540 |
| **IR (Fe^II^-CO) / cm^-1^** | 1969 | 1955 |
| **IR (Fe^II^-CO…CO_3_^2-^) / cm^-1^** | 1678,1957 | Not detected |

**Table S7.** Summary of electrochemical properties of iron porphyrins catalysts for the electrocatalytic CO_2_ reduction.

| **Catalysts** | **Solvent** | **Proton Source** | **η / V** | **log (TOF_max_)** | **FE_CO_ (%)** | **Reference** |
| --- | --- | --- | --- | --- | --- | --- |
| ***α_2_β_2_*-UrFe** | **DMF** | **5.55 M H_2_O** | **0.50** | **6.89** | **84** | **This work** |
| ***αβαβ*-UrFe** | DMF | 5.55 M H_2_O | 0.42 | 3.75 | 91 | ^[10]^ |
| ***αα*-UrFe** | DMF | 2.22 M H_2_O | 0.56 | 4.08 | 91 | ^[4]^ |
| ***αβ*-UrFe** | DMF | 2.22 M H_2_O | 0.61 | 4.71 | 95 | ^[4]^ |
| ***αβαβ*-AmFe** | DMF | 5.55 M H_2_O | 0.66 | 3.85 | - | ^[10]^ |
| **Fe-o-1-amide** | DMF | 0.1 M PhOH | 0.74 | 4.35 | 83 | ^[12]^ |
| **Fe-o-2-amide** | DMF | 0.1 M PhOH | 0.80 | 6.74 | 92 | ^[12]^ |
| **Fe-p-1-amide** | DMF | 0.1 M PhOH | 0.77 | 2.23 | 74 | ^[12]^ |
| **Fe-p-2-amide** | DMF | 0.1 M PhOH | 0.78 | 3.84 | 79 | ^[12]^ |
| **Fe-o-amine** | DMF | 1 M PhOH | 0.76 | 4.02 | 88 | ^[12]^ |
| **Fe-m-amine** | DMF | 1 M PhOH | 0.82 | 3.40 | 87 | ^[12]^ |
| **Fe-AmtBu_4_** | ACN | 3 M PhOH | 0.62 | 5.71 | - | ^[8]^ |
| ***αααα*-FeTMA** | DMF | 3 M PhOH | 0.48 | 5.30 | - | ^[14]^ |
| ***αααβ*-FeTMA** | DMF | 3 M PhOH | 0.37 | 5.00 | - | ^[14]^ |
| ***α_2_β_2_*-FeTMA** | DMF | 3 M PhOH | 0.39 | 5.00 | - | ^[14]^ |
| ***αβαβ*-FeTMA** | DMF | 3 M PhOH | 0.39 | 4.60 | - | ^[14]^ |
| ***αααα-*FeIm** | DMF | 5.55 M H_2_O | 0.23 | 2.49 | 91 | ^[15]^,^[16]^ |
| ***αα*-FeIm** | DMF | 5.55 M H_2_O | 0.43 | 5.93 | 93 | ^[16]^ |
| **FeIm** | DMF | 5.55 M H_2_O | 0.62 | 8.31 | 89 | ^[16]^ |
| **FeImF_6_** | DMF | 5.55 M H_2_O | 0.53 | 5.99 | 92 | ^[16]^ |
| **Fe-o-TMA** | DMF | 3 M PhOH | 0.25 | 6.00 | 93 | ^[17]^ |
| **Fe-p-TMA** | DMF | 3 M PhOH | 0.57 | 4.40 | 92 | ^[17]^ |
| **Fe-p-SA** | DMF | 3 M PhOH | 0.74 | 3.60 | - | ^[17]^ |
| **sc-FeAmCOO** | DMF | 5 M H_2_O | 0.43 | 4.25 | 80 | ^[18]^ |
| **Fe-OH_4_-F_10_** | DMF | 3 M PhOH | 0.59 | 4.00 | 100 | ^[19]^ |
| **Fe-OH_8_** | DMF | 3 M PhOH | 0.66 | 3.80 | 100 | ^[19]^ |
| **Fe-OH_1_** | DMF | 1 M H_2_O | 0.91 | 2.11 | - | ^[20]^ |
| **Fe-OH_1_** | ACN | 1 M H_2_O | 0.78 | 3.7 | 96 | ^[20]^ |
| **Fe-OH_2_** | DMF | 0.1 M PhOH | 0.76 | 6.21 | - | ^[21]^ |
| ***cis*-Fe-OH_4_** | DMF | 0.1 M PhOH | 0.75 | 6.13 | - | ^[21]^ |
| ***trans-*Fe-OH_4_** | DMF | 0.1 M PhOH | 0.73 | 5.51 | - | ^[21]^ |
| **Fe-OH_6_** | DMF | 0.1 M PhOH | 0.75 | 5.71 | - | ^[21]^ |
| **Fe-OH_8_** | DMF | 0.1 M PhOH | 0.73 | 4.45 | - | ^[21]^ |
| **Fe-OH-CF_3_** | ACN | 0.1 M PhOH | 1.67 | 5.31 | - | ^[22]^ |
| **Fe-OH-Br** | ACN | 0.1 M PhOH | 1.66 | 5.59 | - | ^[22]^ |
| **Fe-OH-H** | ACN | 0.1 M PhOH | 1.63 | 5.94 | - | ^[22]^ |
| **Fe-OH-OMe** | ACN | 0.1 M PhOH | 1.66 | 5.54 | - | ^[22]^ |
| **Fe-Cat** | DMF | 1 mM Catechol | 0.84 | 2.57 | 75 | ^[23]^ |
| **TPPFe** | DMF | 5.55 M H_2_O | 0.68 | 4.03 | 94 | ^[16]^ |
| **F_8_Fe** | DMF | 5.55 M H_2_O | 0.56 | 2.20 | - | ^[16]^ |
| **F_20_Fe** | DMF | 5.55 M H_2_O | 0.37 | 1.27 | 96 | ^[16]^ |

**Figure S22.** Structures of the iron porphyrin catalysts included in the performance comparison presented in Table S7.

F. X-Ray Diffraction Crystallography

**Table S8.** Crystallographic data and structure refinement details.

| **Parameters** | ***α_2_β_2_*-UrFe**  **(CCDC 2536618)** |
| --- | --- |
| Empirical Formula | C_84.41_ H_62.83_ F_24_ Fe N_12_ O_11.61_ |
| *M_r_* | 1942.76 |
| Crystal size, mm^3^ | 0.09 x 0.08 x 0.02 |
| Crystal system | monoclinic |
| Space group | *P* 2_1_*/c* |
| a, Å | 14.6860(18) |
| b, Å | 23.269(3) |
| c, Å | 27.318(3) |
| *α*, ° | 90 |
| *β*, ° | 101.022(4) |
| γ, ° | 90 |
| Cell volume, Å^3^ | 9163.3(19) |
| Z ; Z’ | 4 ; 1 |
| T, K | 200(1) |
| Radiation type; wavelength Å | MoK*α* ; 0.71073 |
| F_000_ | 3952 |
| µ, mm^–1^ | 0.277 |
| *θ* range, ° | 1.866 - 26.434 |
| Reflection collected | 231 573 |
| Reflections unique | 18 772 |
| R_int_ | 0.1710 |
| GOF | 1.368 |
| Refl. obs. (*I*>2σ(*I*)) | 7 474 |
| Parameters | 1221 ; 4 |
| wR_2_ (all data) | 0.4785 |
| R value (*I*>2σ(*I*)) | 0.1696 |
| Largest diff. peak and hole (e^-^ Å^-3^) | 1.733 ; -0.865 |

|  |  |
| --- | --- |

**Figure S23.** ORTEP drawing of compound ***α_2_β_2_*-UrFe**. Thermal ellipsoids are shown at the 10% level. Solvent molecules were omitted for clarity except for the water molecule H-bonded to the hydroxyl group as the axial ligand of the iron center.

G. UV-Visible, Nuclear Magnetic Resonance, and Mass Spectra

**Figure S24.** UV-visible spectrum of ***α_2_β_2_*-Ur** (40 µM) in acetone

**Figure S25.** UV-visible spectrum of ***α_2_β_2_*-UrFe** (50 µM) in DMF

**Figure S26.** ^1^H NMR spectra of ***α_2_β_2_*-amino** (full: top, zoom: bottom CDCl_3_, 300 MHz);

**Figure S27.** ^1^H NMR spectra of ***α_2_β_2_*-Ur** (full: top, zoom: bottom (CD_3_)_2_CO, 300 MHz).

**Figure S28.** ^13^C NMR spectrum of ***α_2_β_2_*-Ur** ((CD_3_)_2_CO, 300 MHz).

**Figure S29.** ESI-HR mass spectrum of ***α_2_β_2_*-Ur** (top), showing the experimental and simulated (bottom) isotope pattern.

**Figure S30.** ESI-HR mass spectrum of ***α_2_β_2_*-UrFe** (top), showing the experimental and simulated (bottom) isotope pattern.

H. Computational Details

Density functional theory (DFT) calculations were performed using the Gaussian 16 version C02 program package.^[24]^ OLYP^[25,26]^ functional corrected with D3 dispersion correction^[27]^ was chosen after a benchmark of various functionals detailed below. Geometry optimization were performed using def2svp^[28]^ basis set and the effect of the solvent (DMF) was modeled using the CPCM approach.^[29]^ This level of theory is denoted CPCM(DMF)-D3-OLYP/def2svp and will be referred has L1. The intermediates were optimized as doublet (S=2, D) and quadruplet (S=4, Q) for the formal Fe(I) species and close and open-shell singlet (S=1, S), triplet (S=3, T) and quintet (S=5, Qt) states for the Fe(0) ones, using broken symmetry approach. The stability of the obtained wavefunctions was systematically tested and reoptimized when needed. Geometry optimizations were followed by vibrational frequency analysis to confirm the obtention of minima or saddle points. Single point energy (SPE) was refined using def2tzvpp^[28]^ basis, D3(BJ) dispersion correction^[30]^ and SMD^[31]^ approach for the solvent. This level of theory is denoted SMD(DMF)-D3(BJ)-OLYP/def2tzvp and will be referred has L2. Natural population analysis of charge and spin was performed with NBO 3.1.^[32]^ The energies of all intermediates are provided in Table S14. The Cartesian coordinates and visualization of each structure is available on Figshare: [https.//doi.org/10.6084/m9.figshare.31869178](https://doi.org/10.6084/m9.figshare.31620370). A +1.89 kcal.mol^-1^ concentration correction was applied to take in account the change of standard state from gas phase to solution in non-unimolecular reactions such as CO_2_ coordination.^[33]^

The optimized structures were drawn with CYLview20.^[34]^ The intramolecular Non-Covalent Interactions (NCI) were calculated from the promolecular densities of the fragments within the DFT-optimized structures and integrated on 3 separate ranges using NCIPLOT 4.0.^[35]^ The visualization of the NCI surfaces was performed with VMD^[36]^ (isosurface 0.28, color scale [-3 ; +3]). Buried Volumes (V_bur_) were calculated with SEQCROW^[[28]^ and mapped with ChimeraX 1.11^[37,38]^ with a radius of 4Å and VDW scale of 1.17. The center of the sphere and orientation of the axis is described in the caption of the figures. The percentage of buried volumes calculated by SEQCROW is given for each octant, relatively to the total buried volume within the sphere. Since we wanted to assess the accessibility of a portion of the sphere only, we multiplied these values by respectively 4 and 8 to describe the percentage of buried volumes within the considered quadrants and octants.

The benchmark was designed following the recommendation of Karton and de Olivera.^[39]^ The properties chosen as references are the geometry of *α_2_β_2_****-***UrFe(III)-OH based on its X-ray structure and the experimental potential of the Fe(III/II) redox event at ***α_2_β_2_-*UrFe** and ***αβαβ-*UrFe**, determined by CV in DMF under argon. The choice of the different functionals is based on a study by Morgante and Peverati.^[40]^ Among more than 200 methods tested in this study, five were selected for their performance for the evaluation of the geometry : the pure GGA functionals OPBE^[25,41–43]^ and OLYP,^[25,26]^ the meta-hybrid GGA functional M06L^[44]^ and the hybrid functionals PBE0^[45]^ and B3LYP^[26,46,47]^ with 20 and 25% Hartree-Fock exchange respectively. The same D3 dispersion correction was added to all the functional to facilitate the comparison. We focused our comparison on one side of the porphyrin only, by removing the urea on the other side. PBE0 and M06 functional were eliminated based on large root mean square deviations (Table S8) that reflect an incorrect description of the second coordination sphere arrangement (Figure S38). B3LYP and OLYP performed the best. A closer comparison of relevant geometrical parameters such as bond distances and angles finally led us to choose OLYP (See Table S17).

**Table S9.** Average root mean squared deviation in Å between the calculated (DFT) bond distances with the different functional and the measured ones (X-ray).

|  | **M06** | **OPBE** | **PBE0** | **B3LYP** | **OLYP** |
| --- | --- | --- | --- | --- | --- |
| **All atoms** | 2.597 | 1.695 | 2.875 | 1.656 | 1.658 |
| **Porphyrin ring** | 0.189 | 0.177 | 0.195 | 0.179 | 0.186 |

After optimization of the two systems in their di-reduced “Fe(I)” and tri-reduced “Fe(0)” states using CPCM(DMF)-D3-OLYP/ def2svp level of theory, SPE were calculated using the pure GGA functionals OLYP^[25,26]^, the hybrid PBE0,^[45]^B3LYP,^[26,46,47]^B3PW91,^[46–48]^ and the long-range corrected hybrid wB97XD^[49]^ functionals, with a larger def2tzvpp basis set and including D3(BJ) dispersion correction, except when already included in the functional (w97XD). The stability of the DFT wavefunction was systematically tested and reoptimized when needed. The prediction of standard reduction potentials (SRP) for open-shell first-row transition-metal complexes using DFT is challenging because of significant static correlation and spin-state effects that single-determinant DFT handles imperfectly. In addition, implicit solvation models introduce systematic errors into solvation free energies, especially when the charge and electronic structure differ between the oxidized and reduced states. These problems often lead to large deviations from experimental potentials (up to 1 V) in direct DFT calculations.^[50]^ The isodesmic reaction approach improves on the direct method by computing the potential of a target redox couple relative to a reference redox couple with a known experimental SRP.^[51]^ In this scheme, the systematic errors in DFT-computed free energies cancels out, providing that the reference complex has a similar coordination environment, oxidation states, and ligand type. We applied this methodology for the calculation of the formal Fe^I/0^ SRP of ***α_2_β_2_*-UrFe** and ***αβαβ*-UrFe** using iron tetraphenylporphyrin **TPPFe^I/0^** as a reference (E_1/2_(DMF) = –2.145 V vs Fc^+/0^). Among the 5 tested functionals, OLYP revealed the smallest degree of error for the two atropisomers (Table S17). Besides, the order of the Fe^I/0^ SRP calculated with OLYP for the simplified **^m^*α_2_β_2_*-UrFe** and **^m^*αβαβ*-UrFe** (see Table S17) system follows the same trend as in the real system. This functional was therefore selected for SP refinement in the rest of the study.

**Table S10.** Formal Fe^I/0^ standard redox potential (V) calculated vs Fc^+/0^ for the two atropisomers (without simplification of the structure)

| **Functional** | ***α_2_β_2_*-UrFe** | | ***αβαβ*-UrFe** | | | **Mean Error** |
| --- | --- | --- | --- | --- | --- | --- |
|  | E(Fe^I/0^) | Error | | E(Fe^I/0^) | Error |  |
| PBE0 | -2.38 | 0.49 | | -2.65 | 0.85 | 0.67 |
| B3LYP | -2.35 | 0.46 | | -2.19 | 0.39 | 0.43 |
| B3PW91 | -2.10 | 0.21 | | -2.03 | 0.23 | 0.22 |
| wB97XD | -2.36 | 0.47 | | -1.74 | 0.06 | 0.27 |
| OLYP | -2.01 | 0.12 | | -1.99 | 0.19 | 0.16 |
| *Experimental* | *-1.89* | *-* | | *-1.80* | *-* | *-* |

**Figure S31.** Structures optimized for the formal Fe(I) state of ***αβαβ*-UrFe** (Left) and ***α_2_β_2_*-UrFe** (Right) showing a greater compactness of the former. Of note, the different packing of the SCS in the two atropisomers is in agreement with the measured diffusion coefficients and could play a role by amplifying the 20 mV cathodic shift calculated between ***αβαβ*-UrFe** and ***α_2_β_2_*-UrFe** (see main text).

**Table S11.** Gibbs Free energies change for the first and second addition of a CO_2_ molecule at the two model isomers and **TPPFe** considering the reaction at formal Fe(I) and Fe(0) oxidation states

| **Product** | **ΔG (kcal/mol)** |  | **Product** | **ΔG (kcal/mol)** |
| --- | --- | --- | --- | --- |
| [^m^*αβαβ*-UrFe-CO_2_]^–^ | -6.6 |  | [^m^*αβαβ*-UrFe-(CO_2_)_2_]^–^ | 21.9 |
| [^m^*αβαβ*-UrFe-CO_2_]^2–^ | -20.8 |  | [^m^*αβαβ*-UrFe-(CO_2_)_2_]^2–^ | 17.4 |
| [^m^*α_2_β_2_*-UrFe-CO_2_]^–^ | 2.5 |  | [^m^*α_2_β_2_*-UrFe-(CO_2_)_2_]^–^ | Not stable |
| [^m^*α_2_β_2_*-UrFe-CO_2_]^2–^ | -11.8 |  | [^m^*α_2_β_2_*-UrFe-(CO_2_)_2_]^2–^ | 6.2 |
| [TPPFe-CO_2_]^2–^ | -2.9 |  | [TPPFe-(CO_2_)_2_]^2–^ | 7.3 |

**Figure S32.** Structures optimized for the [Fe-CO_2_]^–^ adducts at **^m^*αβαβ*-UrFe** (Left) and **^m^*α_2_β_2_*-UrFe** (Right). Relevant bond distances and angles are shown in black (hydrogen bonds) and red (Fe-CO_2_ fragment).

**Figure S33.** Structures optimized for the [Fe-CO_2_]^2–^ adducts at **^m^*αβαβ*-UrFe** (Left), **^m^*α_2_β_2_*-UrFe** (Middle) and **TPPFe** (Right). Relevant bond distances and angles are shown in black (hydrogen bonds) and red (Fe-CO_2_ fragment). Greater bending of the O-C-O fragment and lengthening of the C-O bonds reveal a more advanced activation of the CO_2_ fragment in the presence of SCS.

**Table S12.** Natural charges and densities of the CO_2_ fragment in the Fe-CO_2_ intermediates in Fe(I) and Fe(0) formal oxidation states.

| **System** | **Natural Charge at CO_2_** | **Natural Spin density at CO_2_** |
| --- | --- | --- |
| [^m^*αβαβ*-UrFe-CO_2_]^–^ | -0.597 | -0.095 |
| [^m^*αβαβ*-UrFe-CO_2_]^2–^ | -0.702 | 0.035 |
| [^m^*α_2_β_2_*-UrFe-CO_2_]^–^ | -0.572 | -0.092 |
| [^m^*α_2_β_2_*-UrFe-CO_2_]^2–^ | -0.694 | -0.026 |
| [TPPFe-CO_2_]^2–^ | -0.478 | 0.132 |

**Figure S34.** Structures optimized for the [Fe-(CO_2_)_2_]^2–^ adducts at **^m^*αβαβ*-UrFe** (Left), **^m^*α_2_β_2_*-UrFe** (Middle) and **TPPFe** (Right). Relevant bond distances (Å) and dihedral angle (°) are shown in red and gray respectively.

**Table S13.** Comparison of some steric parameters at the [Fe-(CO_2_)_2_]^2–^ adduct for the different Fe porphyrins. The values in bold highlight the greatest differences in **^m^*αβαβ*-UrFe** compared to the two other systems.

| **Angle (°) / Distance (Å)** | **^m^*αβαβ*-UrFe** | **^m^*α_2_β_2_*-UrFe** | **TPP-Fe** |  |
| --- | --- | --- | --- | --- |
| **O2-C1-O3-C4** | **65.3** | 19.0 | 18.7 |  |
| **Fe7-C1-O2** | **128.3** | 125.1 | 123.6 |  |
| **Fe7-C1-O3** | 113.5 | 115.8 | 112.5 |  |
| **O2-C1-O3** | 118.3 | 119.0 | 124.0 |  |
| **C1-O3-C4** | 118.5 | 114.7 | 118.6 |  |
| **O3-C4-O5** | 111.4 | 112.6 | 110.4 |  |
| **O3-C4-O6** | 118.3 | 114.0 | 136.9 |  |
| **O5-C4-O6** | 129.9 | 133.3 | 112.5 |  |
| **Fe7-C1** | 1.83 | 1.84 | 1.90 |  |
| **C1-O3** | **1.39** | 1.35 | 1.34 |  |
| **C1-O2** | 1.23 | 1.25 | 1.23 |  |
| **O3-C4** | **1.42** | 1.51 | 1.56 |  |
| **C4-O6** | 1.26 | 1.25 | 1.23 |  |
| **C4-O5** | 1.24 | 1.23 | 1.23 |  |

**Figure S35.** Structure of the transition state associated with the C–O bond breaking to release CO_3_^2-^ (Left) and the resulting intermediate where CO_3_^2-^ is transiently captured by one urea moiety (Right).

**Figure S36.** Comparison of the energetic profiles for the chemical steps following the formation of the [Fe-CO_2_]^2-^ adduct at the 2 atropisomers and **TPPFe**.

**Table S14.** Characteristic frequencies (in cm^-1^) calculated for carbonyl and carbonate adducts at **^m^α_2_β_2_-UrFe**

| **Theo. vibration mode of** | **v(CO)** | **v(CO_3_^2-^)** |
| --- | --- | --- |
| [Fe(II)-CO_CO_3_]^2-^ | 1948 | 1680 ; 1333 |
| [Fe(II)-CO] | 1979 |  |
| [Fe(I)-CO_3_]^3-^ |  | 1527 ; 1329 |

**Figure S37.** Structure optimized from the geometry of [**^m^*α_2_β_2_*-UrFe-CO_2_]^2-^** where two water molecules were positioned close to the mono-coordinating urea. This structure highlights the role that the SCS in **^m^*α_2_β_2_*-UrFe** might play in organizing protic molecules near the active site.

**Table S15.** Calculated standard reduction potentials vs Fc^0/+^

| **Oxidant** | **Reductant** | **E_Theo_ (V)** |
| --- | --- | --- |
| [^m^*α_2_β_2_*-UrFe]^0^ | [^m^*α_2_β_2_*-UrFe]^-^ | -1.21 |
| [^m^*α_2_β_2_*-UrFe]^-^ | [^m^*α_2_β_2_*-UrFe]^2-^ | -1.92 |
| [^m^*α_2_β_2_*-UrFe-CO_2_]^-^ | [^m^*α_2_β_2_*-UrFe-CO_2_]^2-^ | -1.30 |
| [^m^*α_2_β_2_*-UrFe-CO_CO_3_]^2-^ | [^m^*α_2_β_2_*-UrFe-CO_CO_3_]^3-^ | -1.73 |
| [^m^*α_2_β_2_*-UrFe-CO_CO_3_]^3-^ | [^m^*α_2_β_2_*-UrFe-CO_CO_3_]^4-^ | -2.11 |
| [^m^*α_2_β_2_*-UrFe-CO]^0^ | [^m^*α_2_β_2_*-UrFe-CO]^-^ | -1.36 |
| [^m^*α_2_β_2_*-UrFe-CO]^-^ | [^m^*α_2_β_2_*-UrFe-CO]^2-^ | -1.98 |
| [^m^*α_2_β_2_*-UrFe-CO_3_]^2-^ | [^m^*α_2_β_2_*-UrFe-CO_3_]^3-^ | -1.72 |
|  |  |  |
| [^m^*αβαβ*-UrFe]^-^ | [^m^*αβαβ*-UrFe]^2-^ | -1.83 |
| [^m^*αβαβ*-UrFe-CO_2_]^-^ | [^m^*αβαβ*-UrFe-CO_2_]^2-^ | -1.22 |
| [^m^*αβαβ*-UrFe-CO]^0^ | [^m^*αβαβ*-UrFe-CO]^-^ | -1.35 |
| [^m^*αβαβ*-UrFe-CO]^-^ | [^m^*αβαβ*-UrFe-CO]^2-^ | -1.88 |
|  |  |  |
| [TPPFe]^-^ | [TPPFe]^2-^ | -2.15 |
| [TPPFe-CO]^0^ | [TPPFe^I^-CO]^-^ | -1.76 |
| [TPPFe^I^-CO]^-^ | [TPPFe^0^-CO]^2-^ | -1.82 |
|  |  |  |

**Table S16.** Electronic and Gibbs free energies of each intermediate in their various spin states.

| **Species** | **Charge, q** | **Spin state** | **E_elec_(L1) (au)** | **G(L1) (au)** | **E_elec_(L2) (au)** | **G(L2) (au)** | **ΔG(kcal.mol^-1^)** |
| --- | --- | --- | --- | --- | --- | --- | --- |
| [*αβαβ*-UrFe]^q^ | -1 | Q | -7687.56340 | -7686.60412 | -7695.49990 | -7694.54062 | 19.36 |
|  |  | D | -7687.60118 | -7686.63235 | -7695.54030 | -7694.57147 | 0.00 |
|  | -2 | Qt | -7687.64738 | -7686.69344 | -7695.59775 | -7694.64381 | 6.39 |
|  |  | T | -7687.65985 | -7686.70334 | -7695.61051 | -7694.65400 | 0.00 |
|  |  | S | -7687.66332 | -7686.70282 | -7695.61013 | -7694.64963 | 2.74 |
| [*α_2_β_2_*-UrFe]^q^ | -1 | Q | -7687.57813 | -7686.61143 | -7695.52666 | -7694.55996 | 6.82 |
|  |  | D | -7687.55717 | -7686.60513 | -7695.52287 | -7694.57083 | 0.00 |
|  | -2 | Qt | -7687.63582 | -7686.68513 | -7695.59382 | -7694.64313 | 5.88 |
|  |  | T | -7687.64656 | -7686.69449 | -7695.60457 | -7694.65250 | 0.00 |
|  |  | S | -7687.64932 | -7686.69524 | -7695.59562 | -7694.64153 | 6.88 |
| [^m^*αβαβ*-UrFe]^q^ | -1 | Q | -5431.23077 | -5430.48875 | -5436.45253 | -5435.71051 | 3.49 |
|  |  | D | -5431.24349 | -5430.49640 | -5436.46316 | -5435.71607 | 0.00 |
|  | -2 | Qt | -5431.29314 | -5430.56106 | -5436.52473 | -5435.79265 | 7.43 |
|  |  | T | -5431.30380 | -5430.57124 | -5436.53705 | -5435.80449 | 0.00 |
|  |  | S | -5431.32640 | -5430.58174 | -5436.54343 | -5435.79877 | 3.59 |
| [^m^*α_2_β_2_*-UrFe]^q^ | 0 | T | -5431.119225 | -5430.378781 | -5436.353829 | -5435.613385 | 0.00 |
|  |  | S | -5431.094277 | -5430.352222 | -5436.329994 | -5435.587939 | 15.97 |
|  | -1 | Q | -5431.23385 | -5430.48871 | -5436.45876 | -5435.71362 | 7.07 |
|  |  | D | -5431.23122 | -5430.49171 | -5436.46439 | -5435.72488 | 0.00 |
|  | -2 | Qt | -5431.30653 | -5430.56716 | -5436.52228 | -5435.78291 | 17.06 |
|  |  | T | -5431.327686 | -5430.58647 | -5436.55131 | -5435.81009 | 0.00 |
|  |  | S | -5431.31986 | -5430.57923 | -5436.53849 | -5435.79786 | 7.67 |
| [TPPFe]^q^ | -1 | Q | -3174.87893 | -3174.36621 | -3177.38662 | -3176.87390 | 5.02 |
|  |  | D | -3174.88525 | -3174.37274 | -3177.39441 | -3176.88190 | 0.00 |
|  | -2 | Qt | -3174.94662 | -3174.43764 | -3177.45184 | -3176.94286 | 9.96 |
|  |  | T | -3174.95648 | -3174.44689 | -3177.46832 | -3176.95873 | 0.00 |
|  |  | S | -3174.96460 | -3174.45152 | -3177.47130 | -3176.95822 | 0.32 |
| [^m^*αβαβ*-UrFe-CO_2_]^q^ | -1 | Q | -5619.67867 | -5618.92384 | -5625.11142 | -5624.35659 | 9.95 |
|  |  | D | -5619.68624 | -5618.93454 | -5625.12415 | -5624.37245 | 0.00 |
|  | -2 | Qt | -5619.77356 | -5619.02315 | -5625.20511 | -5624.45470 | 18.03 |
|  |  | T | -5619.79605 | -5619.04593 | -5625.21740 | -5624.46729 | 6.28 |
|  |  | S | -5619.80531 | -5619.04766 | -5625.24109 | -5624.48343 | 0.00 |
| [^m^*α_2_β_2_*-UrFe-CO_2_]^q^ | -1 | Q | -5619.66582 | -5618.91732 | -5625.10611 | -5624.35761 | 5.74 |
|  |  | D | -5619.67962 | -5618.92517 | -5625.12121 | -5624.36676 | 0.00 |
|  | -2 | Qt | -5619.75881 | -5619.01428 | -5625.19722 | -5624.45269 | 13.84 |
|  |  | T | -5619.77145 | -5619.02142 | -5625.21101 | -5624.46097 | 8.64 |
|  |  | S | -5619.78722 | -5619.03462 | -5625.22735 | -5624.47475 | 0.00 |
| [TPPFe-CO_2_]^q^ | -2 | Qt | -3363.36837 | -3362.85035 | -3366.10008 | -3365.58206 | 16.96 |
|  |  | T | -3363.38685 | -3362.86799 | -3366.12231 | -3365.60345 | 3.53 |
|  |  | S | -3363.41599 | -3362.89284 | -3366.13223 | -3365.60908 | 0.00 |
| [^m^*αβαβ*-UrFe-(CO_2_)_2_]^q^ | -1 | Q | *spontaneous decoordination* | | | | *-* |
|  |  | D | -5808.08443 | -5807.31892 | -5813.74893 | -5812.98342 | 0.00 |
|  | -2 | Qt | -5808.68377 | -5807.92311 | -5813.83557 | -5813.07491 | 16.70 |
|  |  | T | -5808.71581 | -5807.95109 | -5813.84889 | -5813.08417 | 10.89 |
|  |  | S | -5808.72490 | -5807.95463 | -5813.87179 | -5813.10152 | 0.00 |
| [^m^*α_2_β_2_*-UrFe-(CO_2_)_2_]^q^ | -2 | Qt | -5808.18067 | -5807.42084 | -5813.83646 | -5813.07663 | 21.37 |
|  |  | T | -5808.18904 | -5807.42671 | -5813.84924 | -5813.08691 | 14.92 |
|  |  | S | -5808.21720 | -5807.45018 | -5813.87770 | -5813.11068 | 0.00 |
| ts1 | -2 | S | -5808.03312 | -5807.26879 | -5813.87289 | -5813.10857 | 0.00 |
| [TPPFe-(CO_2_)_2_]^q^ | -2 | Qt | *spontaneous decoordination* | | | | - |
|  |  | T | *spontaneous decoordination* | | | | - |
|  |  | S | -3551.81810 | -3551.28479 | -3554.77655 | -3554.24324 | 0.00 |
| [^m^*α_2_β_2_*-UrFe-CO_CO_3_]^q^ | -2 | Qt | -5808.15152 | -5807.39807 | -5813.8135 | -5813.06005 | 26.55 |
|  |  | T | -5808.16297 | -5807.40497 | -5813.82872 | -5813.07071 | 19.86 |
|  |  | S | -5808.19618 | -5807.43392 | -5813.86463 | -5813.10236 | 0.00 |
|  | -3 | Q | -5808.27547 | -5807.52214 | -5813.93438 | -5813.18106 | 8.52 |
|  |  | D | -5808.28746 | -5807.52893 | -5813.95317 | -5813.19464 | 0.00 |
|  | -4 | S | -5808.36047 | -5807.60540 | -5814.02801 | -5813.27294 | 0.00 |
| ts2 | -2 | S | -5808.18367 | -5807.42229 | -5813.84656 | -5813.08518 | 0.00 |
| [^m^*αβαβ*-UrFe-CO]^q^ | 0 | Qt | -5544.37330 | -5543.62486 | -5549.69755 | -5548.94911 | 26.00 |
|  |  | T | -5544.38523 | -5543.63183 | -5549.71643 | -5548.96303 | 17.27 |
|  |  | S | -5544.41835 | -5543.66055 | -5549.74835 | -5548.99055 | 0.00 |
|  | -1 | Q | -5544.48357 | -5543.734 | -5549.82659 | -5549.07702 | 12.47 |
|  |  | D | -5544.49316 | -5543.74285 | -5549.84721 | -5549.09689 | 0.00 |
|  | -2 | Qt | -5544.5765 | -5543.83163 | -5549.91749 | -5549.17262 | 6.82 |
|  |  | T | -5544.58102 | -5543.83072 | -5549.93098 | -5549.18068 | 1.77 |
|  |  | S | -5544.57752 | -5543.83014 | -5549.93088 | -5549.18349 | 0.00 |
| [^m^*α_2_β_2_*-UrFe-CO]^q^ | 0 | Qt | -5544.37103 | -5543.62138 | -5549.69733 | -5548.94768 | 25.06 |
|  |  | T | -5544.37882 | -5543.62775 | -5549.70869 | -5548.95763 | 18.82 |
|  |  | S | -5544.41360 | -5543.65553 | -5549.74568 | -5548.98762 | 0.00 |
|  | -1 | Q | -5544.47440 | -5543.72510 | -5549.82426 | -5549.07496 | 11.71 |
|  |  | D | -5544.48471 | -5543.73494 | -5549.84339 | -5549.09361 | 0.00 |
|  | -2 | Qt | -5544.56450 | -5543.82102 | -5549.91255 | -5549.16908 | 4.83 |
|  |  | T | -5544.56947 | -5543.81985 | -5549.92598 | -5549.17636 | 0.27 |
|  |  | S | -5544.56906 | -5543.82041 | -5549.92543 | -5549.17678 | 0.00 |
| [TPPFe-CO]^q^ | 0 | Qt | -3287.98605 | -3287.47021 | -3290.62577 | -3290.10993 | 25.66 |
|  |  | T | -3287.99441 | -3287.47464 | -3290.63899 | -3290.11922 | 19.84 |
|  |  | S | -3288.02730 | -3287.50267 | -3290.67546 | -3290.15083 | 0.00 |
|  | -1 | Q | -3288.11452 | -3287.59905 | -3290.75263 | -3290.23716 | 3.12 |
|  |  | D | -3288.11724 | -3287.59792 | -3290.76145 | -3290.24213 | 0.00 |
|  | -2 | Qt | -3288.19686 | -3287.68539 | -3290.83636 | -3290.32489 | 3.98 |
|  |  | T | -3288.20266 | -3287.68384 | -3290.85006 | -3290.33124 | 0.00 |
|  |  | S | -3288.20226 | -3287.68391 | -3290.84956 | -3290.33121 | 0.02 |
| [^m^*α_2_β_2_*-UrFe-CO_3_]^q^ | -2 | Qt | -5694.98825 | -5694.23628 | -5700.50487 | -5699.75290 | 0.00 |
|  |  | T | -5694.98570 | -5694.23252 | -5700.50601 | -5699.75284 | 0.04 |
|  |  | S | -5694.97002 | -5694.21406 | -5700.49266 | -5699.73669 | 10.17 |
|  | -3 | Q | -5695.06662 | -5694.31807 | -5700.58758 | -5699.83903 | 4.69 |
|  |  | D | -5695.07515 | -5694.32462 | -5700.59704 | -5699.84651 | 0.00 |
| CO_2_ | 0 | S | -188.41328 | -188.42258 | -188.63952 | -188.64882 | - |
| CO_3_^2-^ | -2 | S | -263.71208 | -263.72362 | -264.04753 | -264.05907 | - |
| CO | 0 | S | -113.20105 | -113.21526 | -113.33228 | -113.34649 | - |

**Table S17.** Comparison of some geometrical parameters measured in the X-ray structure and calculated with the different functional tested in the benchmark

| **Distance (Å)** | **X-ray** | **M06L** | **Error** | **OPBE** | **Error** | **PBE0** | **Error** | **B3LYP** | **Error** | **OLYP** | **Error** |
| --- | --- | --- | --- | --- | --- | --- | --- | --- | --- | --- | --- |
| **Fe-OH** | 1.938 | 1.881 | -2.94% | 1.871 | -3.46% | 1.869 | -3.56% | 1.879 | -3.04% | 1.884 | -2.79% |
| **H_2_O-Ur** | 1.988 | 1.945 | -2.16% | 1.722 | -13.38% | 1.742 | -12.37% | 1.758 | -11.57% | 1.803 | -9.31% |
| **Fe-N1** | 1.994 | 2.081 | 4.36% | 2.095 | 5.07% | 2.068 | 3.71% | 2.091 | 4.86% | 2.109 | 5.77% |
| **UrH_up_-(OH)Me** | 2.001 | 1.885 | -5.80% | 1.728 | -13.64% | 2.077 | 3.80% | 1.810 | -9.55% | 1.815 | -9.30% |
| **(HO)-H_2_O** | 2.017 | 1.715 | -14.97% | 1.643 | -18.54% | 1.629 | -19.24% | 1.650 | -18.20% | 1.687 | -16.36% |
| **Fe-N4** | 2.060 | 2.085 | 1.21% | 2.088 | 1.36% | 2.075 | 0.73% | 2.092 | 1.55% | 2.103 | 2.09% |
| **Fe-N2** | 2.108 | 2.073 | -1.66% | 2.097 | -0.52% | 2.073 | -1.66% | 2.098 | -0.47% | 2.108 | 0.00% |
| **Fe-N3** | 2.111 | 2.066 | -2.13% | 2.090 | -0.99% | 2.062 | -2.32% | 2.088 | -1.09% | 2.102 | -0.43% |
| **UrH_down_-(OH)Me** | 2.421 | 2.208 | -8.80% | 2.352 | -2.85% | 1.917 | -20.82% | 2.170 | -10.37% | 2.338 | -3.43% |
| **Angle (°)** | **X-ray** | **M06L** | **Error** | **OPBE** | **Error** | **PBE0** | **Error** | **B3LYP** | **Error** | **OLYP** | **Error** |
| **N3-Fe-N4** | 85.48 | 87.440 | 2.29% | 87.720 | 2.62% | 87.660 | 2.55% | 87.600 | 2.48% | 87.600 | 2.48% |
| **N2-Fe-N3** | 85.72 | 87.770 | 2.39% | 87.410 | 1.97% | 87.960 | 2.61% | 87.610 | 2.20% | 87.190 | 1.71% |
| **N1-Fe-N2** | 87.11 | 87.400 | 0.33% | 87.280 | 0.20% | 87.450 | 0.39% | 87.370 | 0.30% | 87.270 | 0.18% |
| **N4-Fe-N1** | 88.58 | 86.940 | 1.85% | 87.760 | 0.93% | 87.600 | 1.11% | 87.780 | 0.90% | 87.420 | 1.31% |

**Figure S38.** Comparative view of the X-ray structure and the geometries optimized with the different functionals

References

[1]	G. M. Sheldrick, *SHELXS 97, program for the solution of crystal structure*, University Of Göttingen, Germany, **1997**.

[2]	G. M. Sheldrick, “A short history of ıt SHELX” *Acta Cryst. A* **2008**, *64*, 112–122.

[3]	L. J. Farrugia, “ıt WinGX suite for small-molecule single-crystal crystallography” *J. Appl. Crystallogr.* **1999**, *32*, 837–838.

[4]	P. Gotico, L. Roupnel, R. Guillot, M. Sircoglou, W. Leibl, Z. Halime, A. Aukauloo, “Atropisomeric Hydrogen Bonding Control for CO2 Binding and Enhancement of Electrocatalytic Reduction at Iron Porphyrins” *Angewandte Chemie International Edition* **2020**, *59*, 22451–22455.

[5]	C. Costentin, S. Drouet, M. Robert, J.-M. Savéant, “Turnover Numbers, Turnover Frequencies, and Overpotential in Molecular Catalysis of Electrochemical Reactions. Cyclic Voltammetry and Preparative-Scale Electrolysis” *J. Am. Chem. Soc.* **2012**, *134*, 11235–11242.

[6]	C. Costentin, M. Robert, J.-M. Savéant, “Catalysis of the electrochemical reduction of carbon dioxide” *Chem. Soc. Rev.* **2013**, *42*, 2423–2436.

[7]	M. L. Clark, P. L. Cheung, M. Lessio, E. A. Carter, C. P. Kubiak, “Kinetic and Mechanistic Effects of Bipyridine (bpy) Substituent, Labile Ligand, and Brønsted Acid on Electrocatalytic CO2 Reduction by Re(bpy) Complexes” *ACS Catal.* **2018**, *8*, 2021–2029.

[8]	P. Sen, B. Mondal, D. Saha, A. Rana, A. Dey, “Role of 2nd sphere H-bonding residues in tuning the kinetics of CO2 reduction to CO by iron porphyrin complexes” *Dalton Trans.* **2019**, *48*, 5965–5977.

[9]	I. Azcarate, C. Costentin, M. Robert, J.-M. Savéant, “Dissection of Electronic Substituent Effects in Multielectron–Multistep Molecular Catalysis. Electrochemical CO2-to-CO Conversion Catalyzed by Iron Porphyrins” *J. Phys. Chem. C* **2016**, *120*, 28951–28960.

[10]	P. Gotico, B. Boitrel, R. Guillot, M. Sircoglou, A. Quaranta, Z. Halime, W. Leibl, A. Aukauloo, “Second-Sphere Biomimetic Multipoint Hydrogen-Bonding Patterns to Boost CO2 Reduction of Iron Porphyrins” *Angewandte Chemie International Edition* **2019**, *58*, 4504–4509.

[11]	C. Costentin, M. Robert, J.-M. Savéant, “Current Issues in Molecular Catalysis Illustrated by Iron Porphyrins as Catalysts of the CO _2_-to-CO Electrochemical Conversion” *Acc. Chem. Res.* **2015**, *48*, 2996–3006.

[12] E. Nichols, J. S. Derrick, S. K. Nistanaki, P. T. Smith, C. J. Chang,"Positional effects of second-sphere amide pendants on electrochemical CO2 reduction catalyzed by iron porphyrins" *Chem. Sci.* **2018**, *9*, 2952-2960.

[13] G. Liu, Y.-J. Fan, J.-L. Zhang,"Construction of secondary coordination sphere boosts electrochemical CO2 reduction of iron porphyrins" *J. Porphyrins Phthalocyanines* **2020**, *24*, 465-472.

[14]	D. J. Martin, J. M. Mayer, “Oriented Electrostatic Effects on O2 and CO2 Reduction by a Polycationic Iron Porphyrin” *J. Am. Chem. Soc.* **2021**, *143*, 11423–11434.

[15] A. Khadhraoui, P. Gotico, B. Boitrel, W. Leibl, Z. Halime, A. Aukauloo,"Local ionic liquid environment at a modified iron porphyrin catalyst enhances the electrocatalytic performance of CO(2) to CO reduction in water" *Chem. Commun.* **2018**, *54*, 11630-11633.

[16] A. Khadhraoui, P. Gotico, W. Leibl, Z. Halime, A. Aukauloo,"Through-Space Electrostatic Interactions Surpass Classical Through-Bond Electronic Effects in Enhancing CO2 Reduction Performance of Iron Porphyrins" *ChemSusChem* **2021**, *14*, 1308-1315.

[17] I. Azcarate, C. Costentin, M. Robert, J.-M. Savéant,"Through-Space Charge Interaction Substituent Effects in Molecular Catalysis Leading to the Design of the Most Efficient Catalyst of CO2-to-CO Electrochemical Conversion" *J. Am. Chem. Soc.* **2016**, *138*, 16639-16644.

[18] A. Smith, P. Gotico, R. Guillot, S. Le Gac, W. Leibl, A. Aukauloo, B. Boitrel, M. Sircoglou, Z. Halime,"Hinged Carboxylate in the Artificial Distal Pocket of an Iron Porphyrin Enhances CO(2) Electroreduction at Low Overpotential" *Adv Sci* **2025**, *12*, e2500482.

[19] C. Costentin, G. Passard, M. Robert, J. M. Saveant,"Ultraefficient homogeneous catalyst for the CO2-to-CO electrochemical conversion" *Proc Natl Acad Sci U S A* **2014**, *111*, 14990-14994.

[20] S. Sinha, J. J. Warren,"Unexpected Solvent Effect in Electrocatalytic CO2 to CO Conversion Revealed Using Asymmetric Metalloporphyrins" *Inorg. Chem.* **2018**, *57*, 12650-12656.

[21] A. Sonea, K. L. Branch, J. J. Warren,"The Pattern of Hydroxyphenyl-Substitution Influences CO2 Reduction More Strongly than the Number of Hydroxyphenyl Groups in Iron-Porphyrin Electrocatalysts" *ACS Catalysis* **2023**, *13*, 3902-3912.

[22] A. Sonea, N. R. Crudo, J. J. Warren,"Understanding the Interplay of the Brønsted Acidity of Catalyst Ancillary Groups and the Solution Components in Iron-porphyrin-Mediated Carbon Dioxide Reduction" *J. Am. Chem. Soc.* **2024**, *146*, 3721-3731.

[23] [A. Sonea, J. J. Warren,"Pendant Catechol Group Improves the Performance of Iron Porphyrin CO
 Reduction Catalysts" Acs Catalysis 2025, 15, 1444-1454.

[24]	M. J. Frisch, G. W. Trucks, H. B. Schlegel, G. E. Scuseria, M. A. Robb, J. R. Cheeseman, G. Scalmani, V. Barone, G. A. Petersson, H. Nakatsuji, X. Li, M. Caricato, A. V. Marenich, J. Bloino, B. G. Janesko, R. Gomperts, B. Mennucci, H. P. Hratchian, J. V. Ortiz, A. F. Izmaylov, J. L. Sonnenberg, D. Williams-Young, F. Ding, F. Lipparini, F. Egidi, J. Goings, B. Peng, A. Petrone, T. Henderson, D. Ranasinghe, V. G. Zakrzewski, J. Gao, N. Rega, G. Zheng, W. Liang, M. Hada, M. Ehara, K. Toyota, R. Fukuda, J. Hasegawa, M. Ishida, T. Nakajima, Y. Honda, O. Kitao, H. Nakai, T. Vreven, K. Throssell, J. A. Montgomery Jr., J. E. Peralta, F. Ogliaro, M. J. Bearpark, J. J. Heyd, E. N. Brothers, K. N. Kudin, V. N. Staroverov, T. A. Keith, R. Kobayashi, J. Normand, K. Raghavachari, A. P. Rendell, J. C. Burant, S. S. Iyengar, J. Tomasi, M. Cossi, J. M. Millam, M. Klene, C. Adamo, R. Cammi, J. W. Ochterski, R. L. Martin, K. Morokuma, O. Farkas, J. B. Foresman, D. J. Fox **2016**.

[25]	W.-M. Hoe, A. J. Cohen, N. C. Handy, “Assessment of a new local exchange functional OPTX” *Chemical Physics Letters* **2001**, *341*, 319–328.

[26]	C. Lee, W. Yang, R. G. Parr, “Development of the Colle-Salvetti correlation-energy formula into a functional of the electron density” *Phys. Rev. B* **1988**, *37*, 785–789.

[27]	S. Grimme, J. Antony, S. Ehrlich, H. Krieg, “A consistent and accurate ab initio parametrization of density functional dispersion correction (DFT-D) for the 94 elements H-Pu” *The Journal of Chemical Physics* **2010**, *132*, 154104.

[28]	F. Weigend, R. Ahlrichs, “Balanced basis sets of split valence, triple zeta valence and quadruple zeta valence quality for H to Rn: Design and assessment of accuracy” *Phys. Chem. Chem. Phys.* **2005**, *7*, 3297–3305.

[29]	M. Cossi, N. Rega, G. Scalmani, V. Barone, “Energies, structures, and electronic properties of molecules in solution with the C-PCM solvation model” *Journal of Computational Chemistry* **2003**, *24*, 669–681.

[30]	S. Grimme, S. Ehrlich, L. Goerigk, “Effect of the damping function in dispersion corrected density functional theory” *Journal of Computational Chemistry* **2011**, *32*, 1456–1465.

[31]	A. V. Marenich, C. J. Cramer, D. G. Truhlar, “Universal Solvation Model Based on Solute Electron Density and on a Continuum Model of the Solvent Defined by the Bulk Dielectric Constant and Atomic Surface Tensions” *J. Phys. Chem. B* **2009**, *113*, 6378–6396.

[32]	Glendening, E. D., Reed, A. E., Carpenter, J. E., Weinhold, F. (2003) NBO Version 3.1. Gaussian Inc., Pittsburgh.

[33]	M. Bursch, J.-M. Mewes, A. Hansen, S. Grimme, “Best-Practice DFT Protocols for Basic Molecular Computational Chemistry” *Angewandte Chemie International Edition* **2022**, *61*, e202205735.

[34],	“CYLview Visualization Software - Download,” can be found under https://www.cylview.org/download.html(accessed 12 March 2026), **n.d.**

[35]	R. A. Boto, F. Peccati, R. Laplaza, C. Quan, A. Carbone, J.-P. Piquemal, Y. Maday, J. Contreras-Garcı́a, “NCIPLOT4: Fast, Robust, and Quantitative Analysis of Noncovalent Interactions” *J. Chem. Theory Comput.* **2020**, *16*, 4150–4158.

[36]	W. Humphrey, A. Dalke, K. Schulten, “VMD: Visual molecular dynamics” *Journal of Molecular Graphics* **1996**, *14*, 33–38.

[37]	A. J. Schaefer, V. M. Ingman, S. E. Wheeler, “SEQCROW: A ChimeraX bundle to facilitate quantum chemical applications to complex molecular systems” *Journal of Computational Chemistry* **2021**, *42*, 1750–1754.

[38]	V. M. Ingman, A. J. Schaefer, L. R. Andreola, S. E. Wheeler, “QChASM: Quantum chemistry automation and structure manipulation” *WIREs Computational Molecular Science* **2021**, *11*, e1510.

[39]	A. Karton, M. T. de Oliveira, “Good Practices in Database Generation for Benchmarking Density Functional Theory” *WIREs Computational Molecular Science* **2025**, *15*, e1737.

[40]	P. Morgante, R. Peverati, “Comparison of the Performance of Density Functional Methods for the Description of Spin States and Binding Energies of Porphyrins” *Molecules* **2023**, *28*, 3487.

[41]	N. C. HANDY, A. J. COHEN, “Left-right correlation energy” *Molecular Physics* **2001**, *99*, 403–412.

[42]	J. P. Perdew, K. Burke, M. Ernzerhof, “Generalized Gradient Approximation Made Simple” *Phys. Rev. Lett.* **1996**, *77*, 3865–3868.

[43]	J. P. Perdew, K. Burke, M. Ernzerhof, “Generalized Gradient Approximation Made Simple [Phys. Rev. Lett. 77, 3865 (1996)]” *Phys. Rev. Lett.* **1997**, *78*, 1396–1396.

[44]	Y. Zhao, D. G. Truhlar, “A new local density functional for main-group thermochemistry, transition metal bonding, thermochemical kinetics, and noncovalent interactions” *J. Chem. Phys.* **2006**, *125*, 194101.

[45]	C. Adamo, V. Barone, “Toward reliable density functional methods without adjustable parameters: The PBE0 model” *J. Chem. Phys.* **1999**, *110*, 6158–6170.

[46]	A. D. Becke, “Density-functional exchange-energy approximation with correct asymptotic behavior” *Phys. Rev. A* **1988**, *38*, 3098–3100.

[47]	A. D. Becke, “Density‐functional thermochemistry. III. The role of exact exchange” *The Journal of Chemical Physics* **1993**, *98*, 5648–5652.

[48]	J. P. Perdew, J. A. Chevary, S. H. Vosko, K. A. Jackson, M. R. Pederson, D. J. Singh, C. Fiolhais, “Atoms, molecules, solids, and surfaces: Applications of the generalized gradient approximation for exchange and correlation” *Phys. Rev. B* **1992**, *46*, 6671–6687.

[49]	J.-D. Chai, M. Head-Gordon, “Long-range corrected hybrid density functionals with damped atom–atom dispersion corrections” *Phys. Chem. Chem. Phys.* **2008**, *10*, 6615–6620.

[50]	A. V. Marenich, J. Ho, M. L. Coote, C. J. Cramer, D. G. Truhlar, “Computational electrochemistry: prediction of liquid-phase reduction potentials” *Phys. Chem. Chem. Phys.* **2014**, *16*, 15068–15106.

[51]	S. J. Konezny, M. D. Doherty, O. R. Luca, R. H. Crabtree, G. L. Soloveichik, V. S. Batista, “Reduction of Systematic Uncertainty in DFT Redox Potentials of Transition-Metal Complexes” *J. Phys. Chem. C* **2012**, *116*, 6349–6356.
